# Exploring metabolic effects of dipeptide feed media on CHO cell cultures by in silico model-guided flux analysis

**DOI:** 10.1007/s00253-023-12997-0

**Published:** 2024-01-16

**Authors:** Seo-Young Park, Jinsung Song, Dong-Hyuk Choi, Uiseon Park, Hyeran Cho, Bee Hak Hong, Yaron R. Silberberg, Dong-Yup Lee

**Affiliations:** 1 School of Chemical Engineering, Sungkyunkwan University, 2066 Seobu-Ro, Jangan-Gu, Suwon-Si, Gyeonggi-Do 16419 South Korea; 2 Ajinomoto CELLiST Korea Co., Inc., 70 Songdogwahak-Ro, Yeonsu-Gu, Incheon, South Korea

**Keywords:** Chinese hamster ovary cells, Dipeptide, Tyrosine, Feed media formulation, Multivariate statistical analysis, Flux balance analysis

## Abstract

**Abstract:**

There is a growing interest in perfusion or continuous processes to achieve higher productivity of biopharmaceuticals in mammalian cell culture, specifically Chinese hamster ovary (CHO) cells, towards advanced biomanufacturing. These intensified bioprocesses highly require concentrated feed media in order to counteract their dilution effects. However, designing such condensed media formulation poses several challenges, particularly regarding the stability and solubility of specific amino acids. To address the difficulty and complexity in relevant media development, the biopharmaceutical industry has recently suggested forming dipeptides by combining one from problematic amino acids with selected pairs to compensate for limitations. In this study, we combined one of the lead amino acids, L-tyrosine, which is known for its poor solubility in water due to its aromatic ring and hydroxyl group, with glycine as the partner, thus forming glycyl-L-tyrosine (GY) dipeptide. Subsequently, we investigated the utilization of GY dipeptide during fed-batch cultures of IgG-producing CHO cells, by changing its concentrations (0.125 × , 0.25 × , 0.5 × , 1.0 × , and 2.0 ×). Multivariate statistical analysis of culture profiles was then conducted to identify and correlate the most significant nutrients with the production, followed by in silico model-guided analysis to systematically evaluate their effects on the culture performance, and elucidate metabolic states and cellular behaviors. As such, it allowed us to explain how the cells can more efficiently utilize GY dipeptide with respect to the balance of cofactor regeneration and energy distribution for the required biomass and protein synthesis. For example, our analysis results uncovered specific amino acids (Asn and Gln) and the 0.5 × GY dipeptide in the feed medium synergistically alleviated the metabolic bottleneck, resulting in enhanced IgG titer and productivity. In the validation experiments, we tested and observed that lower levels of Asn and Gln led to decreased secretion of toxic metabolites, enhanced longevity, and elevated specific cell growth and titer.

**Key points:**

• *Explored the optimal Tyr dipeptide for the enhanced CHO cell culture performance*

• *Systematically analyzed effects of dipeptide media by model-guided approach*

• *Uncovered synergistic metabolic utilization of amino acids with dipeptide*

**Graphical abstract:**

**Figure:**
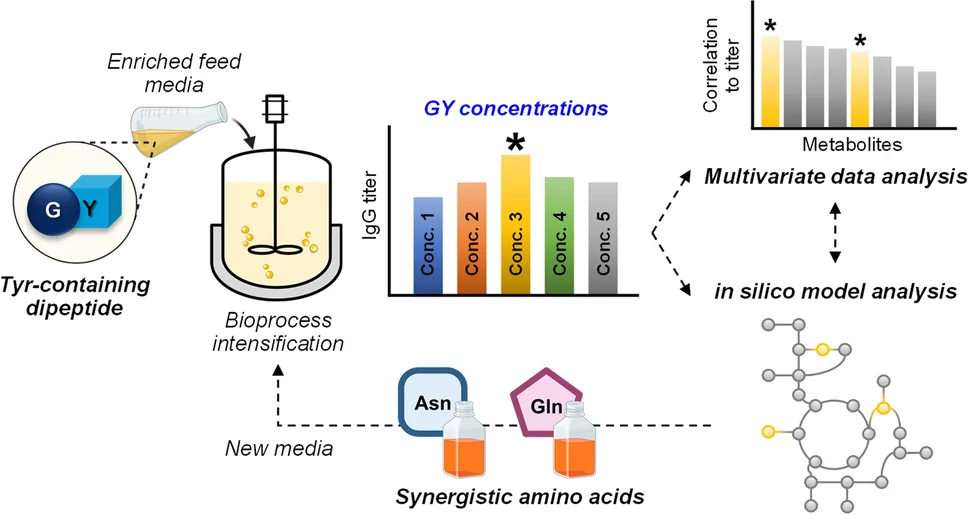

**Supplementary Information:**

The online version contains supplementary material available at 10.1007/s00253-023-12997-0.

## Introduction

Chinese hamster ovary (CHO) cells are renowned for their ability to produce recombinant proteins, making them the preferred mammalian cell factories for commercially manufacturing a wide range of biotherapeutics such as monoclonal antibodies (mAbs) (Park et al. 2021). Over the past few decades, the successful production of biotherapeutics using CHO cells has been realized through the seamless integration of cell line engineering and process optimization. An important aspect of process optimization for CHO cell culture is medium optimization, which significantly contributes to maximizing cell growth, productivity, and product quality (Fouladiha et al. 2020; Yeo et al. 2022). This is achieved through the meticulous calibration of cellular nutritional demands, involving the precise modulation of constituents within the cell culture media—encompassing a spectrum of nutrient concentrations, specific growth factors, and supplements—all of which ensure the provisioning of ample nutrients and energy sources (Kishishita et al. 2015). Furthermore, while fed-batch processes persist as the forefront approach in biopharmaceutical manufacturing, perfusion or continuous processes are being increasingly favored to elevate productivity (Yongky et al. 2019; Xu et al. 2020). These intensified biopharma processes require highly concentrated feeds or media for nutrient replenishment, serving to prevent any reduction in productivity due to dilution effects (Zhang et al. 2020). However, formulating such concentrated feeds or media is challenging owing to the instability (e.g., glutamine) and limited solubility of specific amino acids (e.g., cystine and tyrosine) as well as other critical components (e.g., keto acids) (Christie and Butler 1994; Tabata and Hashimoto 2007; Kang et al. 2012; Nishino et al. 2022). To overcome these challenges, dipeptides, which are formed by combining the troublesome amino acid with another amino acid, are now rapidly being adopted by the biopharmaceutical industry (Kang et al. 2012; Mitsuhashi 2014; Li et al. 2018).

One of the amino acids posing challenges is L-tyrosine (Tyr, Y), known for its poor solubility in water at a neutral pH and room temperature (Lee et al. 2013; He et al. 2018). This is primarily due to the specific structure and properties of its side chain, which contains a hydroxyl group (‒OH) and an aromatic ring. This aromatic ring in L-Tyr contains a large number of hydrophobic carbon atoms that offset the hydrogen bonding interactions formed by the hydroxyl group with water molecules, resulting in limited solubility in aqueous solutions. L-Tyr plays a crucial role as an indispensable amino acid that regulates cellular metabolism, protein synthesis, and the overall quality of products in CHO cell culture (Yu et al. 2011; Feeney et al. 2013; Zhang et al. 2020). In traditional industrialized biomanufacturing, a separate L-Tyr stock solution, adjusted to an extreme alkaline pH to elevate it away from its isoelectric point (pI = 5.64), has been used in addition to the primary pH-neutral feed medium (Lee et al. 2013; Zimmer et al. 2014). However, this method is accompanied by several drawbacks such as increased process complexity and contamination risk, the potential for pH spikes, precipitation risks at elevated concentrations, and the introduction of higher salt levels (Hoang et al. 2021). For this reason, Tyr-conjugated dipeptides have been proposed for use in CHO cell culture media. These dipeptides significantly enhance L-Tyr solubility in water at a neutral pH by up to 75 times compared to free L-Tyr (Fürst 1998), thus possibly enabling a more accurate control over its concentration with fewer preparation steps. This adaptation aligns with a key market trend, meeting the demands of biologics biomanufacturing in terms of the concepts of process simplification and intensification. In this regard, various Tyr-containing dipeptides were investigated to demonstrate their beneficial effects in reducing the formation of toxic byproducts and increasing cell viability and titer (Kang et al. 2012; Sánchez-Kopper et al. 2016; Verhagen et al. 2020). However, the supplementation of Tyr-containing dipeptides in the media increases production costs primarily due to the high expense involved in synthesizing short peptides. Furthermore, our understanding of how dipeptides influence CHO cell culture and subsequent cellular responses remains limited. This knowledge gap impedes the rational design of culture media with optimum dipeptide concentrations.

Thus, further investigation is imperative to unveil their utilization and intricately interconnected cellular metabolism, which motivated us to explore and characterize the metabolic utilization of dipeptide by CHO cells during fed-batch culture. In this work, we initially assessed the impact of substituting L-Tyr with a dipeptide in the feed media on CHO cell performance. Specifically, we evaluated the effectiveness of using selected glycyl-L-tyrosine (GY) dipeptide, known for its cost-effectiveness and ability to enhance solubility, thus facilitating the preparation of concentrated feeds without requiring pH adjustments. Since optimal essential nutrition at the right concentration within the feed medium is pivotal for meeting cellular requirements and ensuring efficient and cost-effective bioprocesses, we systematically examined the effects of varying GY concentrations (0.125 × , 0.25 × , 0.5 × , 1.0 × , and 2.0 ×) on culture performance and cellular responses. In this study, we used our previously established systematic framework where multivariate statistical and in silico model-guided analyses are combined (Hong et al. 2022; Yeo et al. 2022; Park et al. 2023). Briefly, multivariate statistical analysis was conducted to identify metabolites that showed either positive or negative correlations with process productivity, followed by flux balance analysis of a CHO genome-scale metabolic network model to systematically unravel the underlying metabolic mechanisms involved in response to varying GY concentrations.

## Materials and methods

### CHO cell, media, and cell culture

**Fig. 1:**
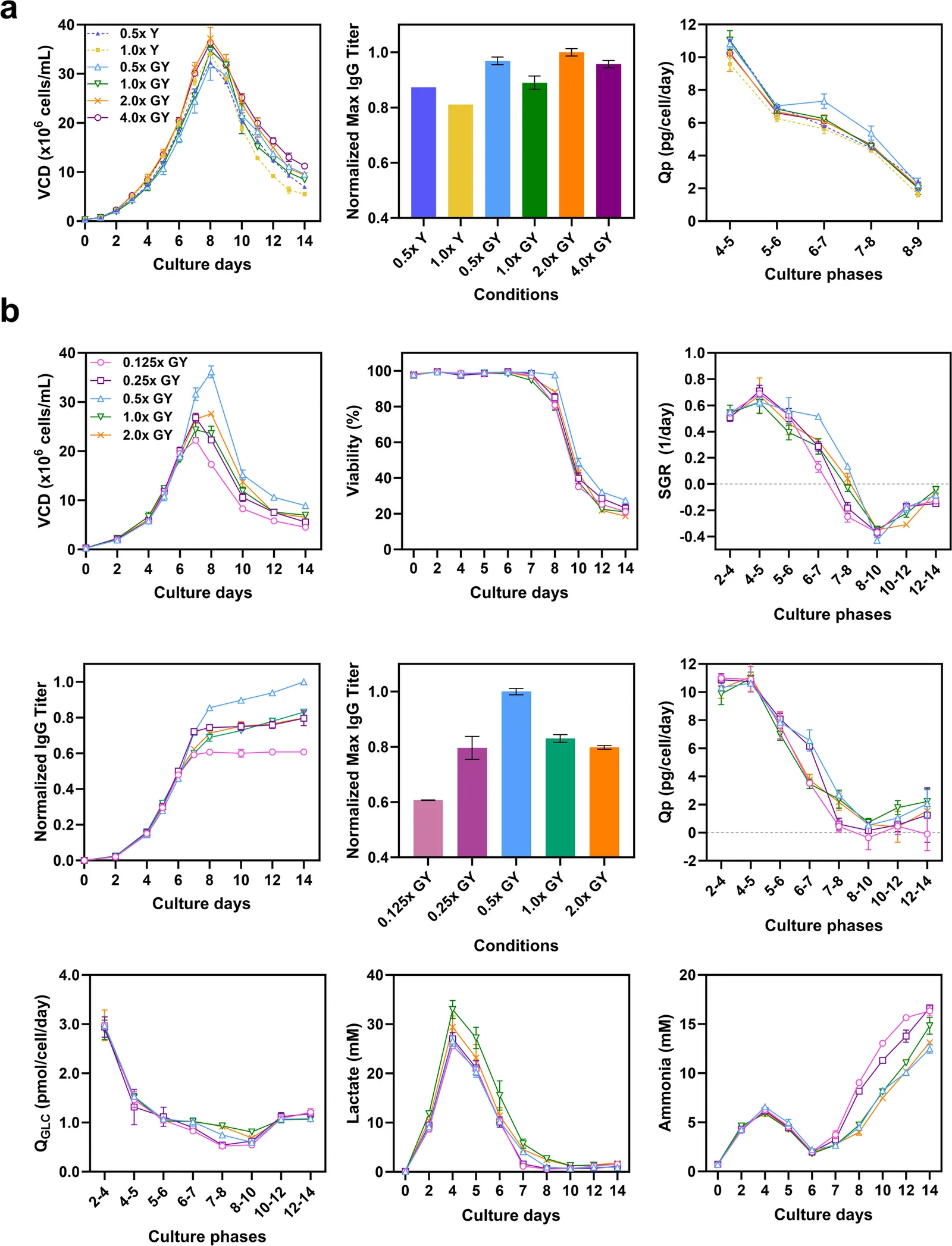
**a** Preliminary fed-batch culture profiles with the addition of tyrosine as monomer L-Tyr (Y, dotted lines) and glycyl-L-Try (GY, solid lines) dipeptide. Viable cell density (VCD), maximum titer, specific IgG production rate (Qp). **b** CHO cell culture profiles for 14 days with varying GY concentrations in the feed media. Each condition was conducted as duplicates or triplicates, standard deviation was drawn as an error bar in the following graphs: VCD, viability, specific growth rate (SGR), titer, maximum titer, Qp, specific glucose consumption rate (Q_GLC_), concentration of lactate and ammonia. Error bars represent the standard deviations of biological duplicates or triplicates. In instances where the error bars are shorter than the size of the symbol, they may not be visibly distinguishable

Chemically defined basal and feed media (CELLiST™, Ajinomoto CELLiST Korea, Co., Inc.) and a proprietary IgG1 antibody-producing CHO-K1 cell line (Ajinomoto CELLiST Korea, Co., Inc.) were used in all experiments. Unless otherwise specified, all chemicals and reagents were purchased from Sigma-Aldrich (St. Louis, MO, USA). Seed expansions for the CHO cell line were operated in 125 mL shake flasks (Corning Life Sciences, NY, USA) for three passages. The shake flasks were placed in a shaking incubator and controlled at 115 rpm, 37 ℃, and 5% CO_2_ humidified atmosphere_._ The cells were cultured in 30 mL of the basal medium at an inoculation density of 3 × 10^5^ cells/mL, supplemented with 6 mM of L-glutamine (Thermo Fisher, Waltham, MA, USA), 5 mg/L of insulin, and 30 μg/mL of puromycin (Thermo Fisher, Waltham, MA, USA). Fed-batch triplicate cultures were performed in ambr15® minibioreactors (Sartorius, Göttingen, Germany). The bioreactors were incubated at 3 × 10^5^ cells/mL in 12 mL of initial working volume and cultured for 14 days under various concentrations of glycyl-L-tyrosine (GY) dipeptide (0.125 × , 0.25 × , 0.5 × , 1 × , 2 × and 4 ×) contained feed media or two different concentrations of separated L-tyrosine stock (pH 11.01) solution (0.5 × and 1 ×) used in addition to the main pH neutral feed. Concentration of 1 × indicates the equivalent mole concentration of tyrosine contained in chemically defined proprietary feed media. Feeding at 4% (v/v) of the current culture volume began on day 4 and was supplemented every 2 days until the end of the culture. Temperature was kept at 37 °C, and dissolved oxygen (DO) level was maintained at 50%. pH was controlled at pH 7.0 ± 0.05 using CO_2_ gas and sodium carbonate. Stir speed was controlled at 1200 rpm. A total of 240 μL of Antifoam C Emulsion was utilized to control overflowing foam. Glucose was added in bolus feeding, reaching up to 6 g/L from day 4 to 8, and increasing to 7 g/L from day 9 to 12, when the remaining glucose concentration fell below the proprietary level. However, in the preliminary experiment (shown in Fig. 1a), a different glucose feeding strategy was employed, maintaining a setpoint of 7 g/L on day 7, with none added on day 11.

### Off-line measurements

Viable cell density (VCD) and cell viability were analyzed using an automatic Cedex HiRes Analyzer (Roche, Basel, Switzerland) with daily samples. Concentrations of glucose, lactate, ammonia, glutamine, glutamate, and IgG were quantified using a Cedex Bio HT Analyzer (Roche, Basel, Switzerland) with samples collected on days 0, 2, 4, 5, 6, 7, 8, 10, 12, and 14. The IgG titer was subsequently normalized relative to the maximum final titer achieved by all cultures. For amino acids and their associated components in the supernatant, the AccQ-Tag Ultra Derivatization kit (Waters, Milford, MA, USA) was employed according to the manufacturer’s instructions. The derivatized samples were analyzed using an ultra-high-performance liquid chromatography system (ACQUITY H-class UPLC, Waters, Milford, USA) equipped with a C18 column (AccQ-Tag ultra, 1.7 μm, 2.1 × 100 mm, Waters, Milford, MA, USA). Amino acid separation occurred on the column at a temperature of 43 ℃, following a gradient-based method. Mobile phase A comprised 100% eluent A (AccQ-Tag Ultra, Waters, Milford, MA, USA), while mobile phase B consisted of a 0.1% (v/v) eluent B (AccQ-Tag Ultra, Waters, Milford, MA, USA) in water. Additionally, mobile phase C was constituted of 100% UPLC grade water, and mobile phase D consisted of 100% eluent B. The gradients profiles of the mobile solutions were described previously (Park et al. 2023). A constant flow rate of 0.7 mL/min was maintained, and a tunable UV (TUV) detector (Acquity UPLC®, Waters, Milford, USA) was set to 260 nm.

### Multivariate data analysis of fed-batch culture data

The culture profiles from triplicated fed-batch run under the culture conditions were combined into a multivariate dataset to identify potential outliers and assess the degree of similarity or dissimilarity among observations for each culture condition. A multivariate dataset comprises daily measured variables (VCD, viability, pH, and DO) as well as metabolite concentrations (glucose, lactate, ammonia, and 20 amino acids) acquired from all sampling timepoints (days 0, 2, 4, 5, 6, 7, 8, 10, 12, and 14), and it was analyzed using SIMCA software (v16.0, Sartorius-Stedim, Umea, Sweden). Prior to conducting the multivariate data analysis (MVDA), an element balance was verified to ensure the integrity of the profiled data and to address issues before they escalate. The collected time profiles were transformed into specific growth rate (SGR, 1/day), integrated viable cell density (IVCD, cell∙day/mL), specific IgG production rate (Qp, pg/cell/day), and specific consumption/production rates of metabolites (Qm, pmol/cell/day) for each phase (phases 0–2, 2–4, 4–5, 5–6, 6–7, 7–8, 8–10, 10–12, 12–14) using the following formula:

1 $${IVCD}_{t\mathrm{1,2}}= \frac{({VCD}_{1}+{VCD}_{2})}{2}\times ({{\mathrm{t}}}_{2}-{{\mathrm{t}}}_{1})$$

2 $${Q}_{m}= \frac{{C}_{2}-({C}_{1}+{C}_{feed,1})}{{IVCD}_{t\mathrm{1,2}}}$$

3 $${Q}_{p}= \frac{{C}_{p, 2}-{C}_{p, 1}}{{IVCD}_{t\mathrm{1,2}}}$$

where ${C}_{p, i}$ and ${C}_{ i}$ are the concentrations of IgG and metabolites, respectively, in residual media at timepoint *i*; ${C}_{feed, i}$ is the elevated concentration of the metabolite resulting from the addition of feed media at timepoint *i*.

Subsequently, an initial data quality check was conducted to identify outliers among triplicate batches (bioreactors). Batches with VCD trends deviating more than one standard deviation from either + 1 or − 1 standard deviation line, unlike other replicated batches, were considered outliers (Figure S2). As a result, 0.125 × GY batch #3, 0.5 × GY batch #1, and 2.0 × GY batch #3 were excluded from the dataset for further analysis. The qualified dataset was then used as input to a batch evolution model (BEM) incorporating all variables (i.e., VCD, viability, IgG, and metabolite concentrations) within a unified data matrix, and a batch-level model (BLM) was derived by decomposing the BEM in order to investigate the impact of various GY concentrations as individual batches at each post-feeding timepoint (days 4, 5, 6, 7, 8, 10, 12, and 14). The BLM-based partial least squares (PLS) analysis was conducted to identify correlations between input *X* variables (metabolite concentrations) and output (*Y*) variable (the final IgG concentration). To determine the importance of the X-variables in varying GY batches on *Y*, metabolites with a variable influence on projection (VIP) value ≥ 1.00, as derived from the PLS analysis, are considered statistically significant in their impact on the final IgG concentration. Coefficient correlation matrices were also generated from the PLS analysis, which represents the relative positive or negative correlations between *X* and *Y*. The sum of coefficient values at each timepoint, for variables with VIP scores ≥ 1.00, was calculated and the resultant values were used to rank the variables based on their positive or negative consistency with the final titer.

### ecFBA with CHO-GEM

To explore how varying GY concentrations in the feed media influence metabolic flux changes and to identify which cellular metabolic pathways are crucial for these differences, we employed the latest CHO genome-scale metabolic model (GEM), *i*CHO2291. We used enzyme-capacity constrained flux balance analysis (ecFBA), which incorporates both cell-specific rates (glucose, lactate, ammonia, amino acids, productivity) and enzyme kinetic parameters (*k*_cat_) as described by Yeo et al. (2020). In the *i*CHO2291 model, we introduced components for GY metabolism, including GY uptake (“EX_glytyr(e)”: ‘glytyr[e] ↔ ’), transport (“GLYTYRPEPT1t”: ‘glytyr[e] ↔ glytyr[c]’), and the cleavage pathway (“GLYTYRHYDRO”: ‘h2o[c] + glytyr[c] → gly[c] + tyr_L[c]’). ecFBA was performed utilizing the constraint-based reconstruction and analysis (COBRA) toolbox v3.0 (Heirendt et al. 2019). The implementation was performed using MATLAB (R2020a, MathWorks), and the linear programming problems were solved using Gurobi (v9.1.1, http://www.gurobi.com) as the solver. Specifically, we conducted ecFBA to describe the exponential growth phase, spanning from day 6 and day 7 after feeding, as this culture phase showed the most distinct SGR and Qp under various GY conditions. The objective function is to maximize biomass production, which refers to the synthesis of cellular components (e.g., DNA, RNA, proteins, carbohydrates, lipids) required for cell growth while fixing the measured production rate of antibody. Note that antibody equation comprises amino acids compositions derived based on the sequence information (serine 13.25 mol%, valine 8.89 mol%, threonine 8.28 mol%, lysine 7.38 mol%, proline 7.23 mol%, leucine 6.87 mol%, glycine 6.63 mol%, alanine 5.87 mol%, glutamate 4.67 mol%, tyrosine 4.67 mol%, glutamine 4.52 mol%, asparagine 3.92 mol%, aspartate 3.46 mol%, phenylalanine 3.01 mol%, cysteine 2.41 mol%, arginine 2.11 mol%, isoleucine 2.11 mol%, histidine 1.96 mol%, tryptophan 1.96 mol%, methionine 0.90 mol%). To account for experimental perturbations and resulting mathematical infeasible solution space, measured uptake and secretion rates of nutrients were relaxed by ± 20% and were contained as upper and lower bounds. To discern the metabolic differences in the flux of reaction i (${v}_{i}$), we calculated fold-change (*FC*) values between 0.125 × (low-GY) and 1.0 × (high-GY) conditions in comparison to 0.5 × (optimum-GY) condition: ${v}_{i,0.125\times {\mathrm{GY}}}$ *vs.* ${v}_{i,0.5\times {\mathrm{GY}}}$ and ${v}_{i,1.0\times {\mathrm{GY}}}$ *vs.*${v}_{i,0.5\times {\mathrm{GY}}}$. Then, we excluded reactions with flux values below 0.0001 mmol/gDCW/h. Subsequently, we explored the most distinctive metabolic reactions based on criteria of *FC* values greater than 1.2.

### Validation experiment

To investigate the effects of target amino acids, increased or decreased concentration of each component was formulated for new experiment sets. Asparagine (Asn) was 30% subtracted from control feed media and another condition with 50% reduced arginine (Arg) concentration was also conducted. Glutamine (Gln) level was decreased to 2 mM in basal media, otherwise other validation experiments were supplemented with 6 mM Gln in the basal media. Validation runs were made into duplicates for each condition and all batches were conducted under the same conditions as the previous run.

## Results

### Effects of different concentrations of GY dipeptide on fed-batch culture performance

A preliminary set of experiments was conducted in fed-batch mode using the IgG1-producing CHO-K1 cell line to assess the impact of substituting free Tyr with dipeptide on cell growth, titer, and productivity. L-Tyr monomer (Y) was tested at concentrations of 0.5 × and 1.0 × , whereas glycyl-L-Tyr dipeptide (GY) was evaluated at concentrations of (0.5 × , 1.0 × , 2.0 × , and 4.0 ×) in the feed media. The resultant culture profiles, including VCD, titer, and Qp, are shown in Fig. 1a. The overall trend in cell growth remained consistent across all culture conditions with cells proliferating from day 2 and reaching their peak VCD (pVCD) on day 8, followed by a subsequent decline. The cultures supplemented with GY achieved enhanced cell growth and titers compared to those supplemented with Y, as expected. On day 8, elevating the Y concentration gave a rise to a 5.26% increase in observed pVCD. However, the VCD declined more rapidly thereafter, leading to a 7.10% lower normalized final titer in the 1.0 × Y condition. Regarding GY supplementation, the pVCD increased as the GY concentration rose from 0.5 × to 2.0 × . However, a similar pVCD was observed between 4.0 × GY (36.3 × 10^6^ cells/mL) and 2.0 × GY (37.2 × 10^6^ cells/mL). The 0.5 × GY condition showed the lowest pVCD among all GY conditions but achieved the highest Qp, particularly between day 6 and day 7. Interestingly, the normalized final titer did not increase gradually when the GY concentration was increased, implying the presence of a suitable GY concentration specifically tailored to our cell line and culture process. These observations prompted us to delve into the optimal GY concentrations and the potential implications of reducing GY concentration below 0.5 × in the feed medium for culture performance.

Subsequently, CHO cell cultures (*N* = 3) were conducted using newly formulated feed media containing varying concentrations of GY (0.125 × , 0.25 × , 0.5 × , 1.0 × , and 2.0 ×) to further evaluate their influence on culture performance (Fig. 1b). The incremental concentrations of GY did not demonstrate a consistent pattern in the culture profiles. Notably, the 0.5 × GY condition exhibited significantly improved VCD, viability, specific growth rate (SGR), and titer. On day 8, the 0.5 × GY condition reached the highest pVCD of 36.15 × 10^6^ cells/mL, surpassing the pVCDs of 26.55 and 24.33 × 10^6^ cells/mL observed in the 0.25 × and 1.0 × GY conditions, respectively. Additionally, the 0.5 × GY condition achieved the highest final titer, while the 0.25 × , 1.0 × , and 2.0 × conditions showed relatively lower final titers of 25.7%, 20.5%, and 25.24%, respectively. Most conditions displayed consistent Qp, except for the 0.5 × GY condition, indicating a strong relationship between the higher SGR observed during the exponential phase and enhanced productivity, which is in agreement with previous observations (Verhagen et al. 2020). In fact, the most effective condition, 0.5 × GY, showed significantly reduced levels of toxic byproducts such as lactate and ammonia throughout the culture duration, fostering a more favorable environment conducive to cell growth. On the contrary, the 0.125 × GY condition resulted in depleted culture profiles, manifesting poor growth and titer (64.65% lower than 0.5 × GY), alongside the highest accumulation of ammonia observed during the culture. Lactate concentrations initially increased until day 4, followed by a subsequent decrease. Regardless of whether the GY concentration in the feed increased or decreased compared to the 1.0 × GY condition, consistently lower lactate concentrations were observed throughout the culture period (0.125 × : 24.15%, 0.25 × : 16.03%, 0.5 × : 22.01%, 2.0 × : 6.27% lower than 1.0 × on day 4).

**Fig. 2:**
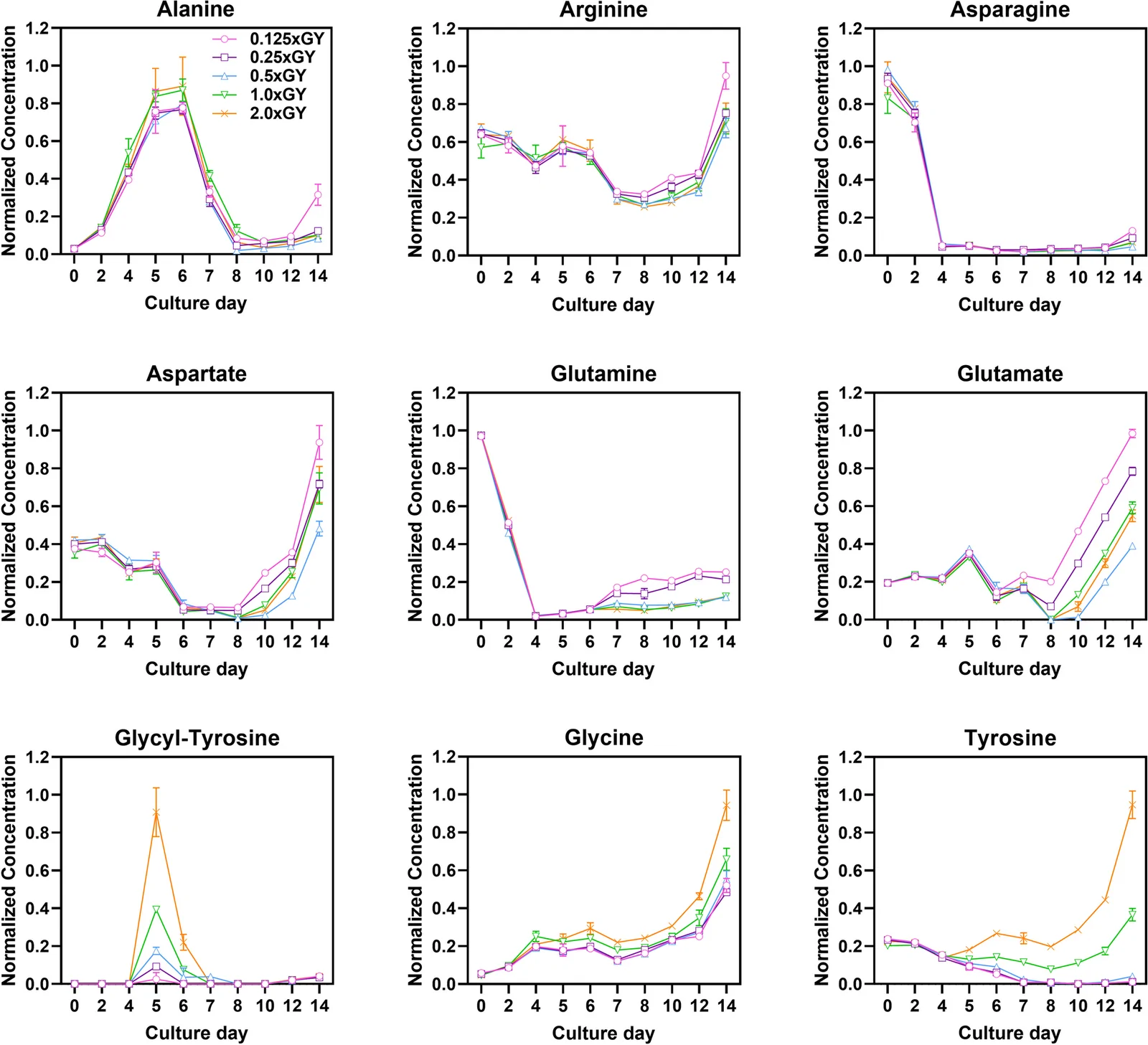
Profiles of residual concentration of amino acids in each culture condition. Amino acid concentrations were normalized to the maximum value among all conditions. Error bars represent the standard deviations of biological duplicates or triplicates. In instances where the error bars are shorter than the size of the symbol, they may not be visibly distinguishable

Moreover, Fig. 2 illustrates the most distinctive profiles of residual amino acid concentrations within the feed media. Alanine was more prominently formulated during the growth phases in higher GY conditions, whereas glutamine and glutamate were increased concentrations during the declining growth phases following the pVCD on day 7 or 8 in lower GY conditions compared to the optimal 0.5 × GY condition. However, asparagine was depleted before feeding in all conditions, while aspartate showed depletion throughout the culture duration until day 8, after which a shift in the trend occurred. In conditions where the GY concentration exceeded that of 0.5 × GY, substantial amounts of residual glycine and tyrosine remained in the culture media, implying that GY dipeptides underwent cleavage into their constitutive amino acids, which were subsequently metabolized. Furthermore, any leftover amino acids that went unused were released outside the cells, a phenomenon reported in the previous study (Sánchez-Kopper et al. 2016). In the pursuit of comprehending the complex relationship between amino acids (Figure S1) and titer, a multivariate data analysis (MVDA) was performed in the downstream analyses. This investigation not only aimed to identify the metabolite profile significantly influenced by different GY conditions but also sought to reveal components consistently associated with higher final titer conditions. This exploration holds the potential to uncover synergetic components that could be strategically utilized in formulating new feed media.

### Multivariate data analysis of culture profiles under various GY levels to identify key nutrients for enhanced IgG production

Initially, we detected and removed outliers among the triplicate batches for each condition. Note that any batch data displaying a VCD trend significantly skewed to one side of either the + 1 or − 1 standard deviation line, unlike other replicated batches, was considered an outlier. Resultantly, 0.125 × GY batch #3, 0.5 × GY batch #1, and 2.0 × GY batch #3 were excluded from the dataset for further analysis (Figure S2). The qualified dataset was then decomposed into a batch-level model (BLM) with time-series observations per batch, allowing us to visualize batch data as trends and identify abnormal data. Additionally, it allows for the identification of critical observations that have the most significant impact on trend changes. To determine which critical observation predominantly influences the most crucial process outcomes, i.e., final titer, PLS regression analysis was conducted between the final titer and the time-series input variables (VCD, viability, concentration of IgG and metabolites on days 4, 5, 6, 7, 8, 10, 12, and 14) across various batches (0.125 × , 0.25 × , 0.5 × , 1.0 × , 2.0 × GY conditions).

**Fig. 3:**
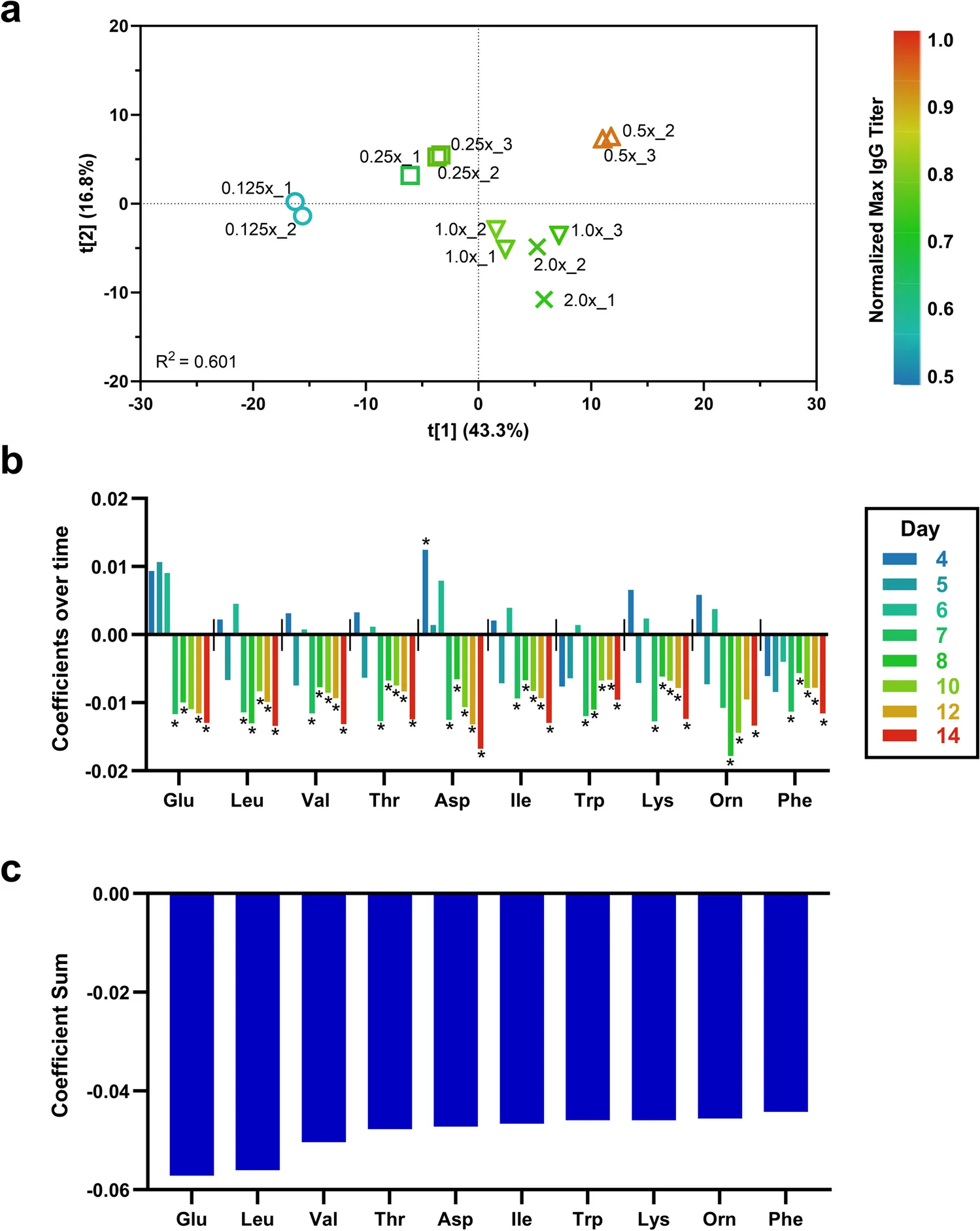
**a** The score plot resulting from the batch level model (BLM)-based PLS analysis. The outlier batch was eliminated from triplicated bioreactors of each GY condition, duplicates or triplicates of batches cluster together on the plot. Variations in GY concentrations within the feed are associated with distinct improvements in maximum titer at the final culture day, as reflected in the coloration of bioreactors (batches)-bluish colors indicating lower titer and reddish colors indicating higher titer. **b** Coefficients for the top 10 statistically important amino acids in feed components at each timepoint during the post-feeding phases (days 4–14) are presented, indicating their correlations with the final titer as positive or negative. Coefficients with a VIP score greater than 1.00 are denoted with an asterisk (*). **c** The sum of coefficients for the top 10 statistically significant amino acids, showing negative correlations with the final titer

The score plot from the PLS model shows that duplicate or triplicate batches (bioreactors) within each GY condition tend to cluster together, indicating a high degree of similarity among bioreactors in each culture condition (Fig. 3a). Furthermore, the plot demonstrates distinct differences in the maximum titer achieved at the final culture day due to varying concentrations of GY in the feed. The coloration of each bioreactor on the plot corresponds to this distinction, with bluish and reddish tones indicating lower and higher titers, respectively. Notably, the group of batches in the 0.5 × GY condition exhibits the highest titer among all groups, underscoring the positive impact of adding 0.5 × GY in the feed-on titer in comparison to other GY concentrations. Thus, to ascertain the metabolites significantly influencing the final titer, the coefficient-sum (Table S1) was calculated by summing the coefficients of metabolites from each timepoint that exhibited a high VIP score (≥ 1.00, Table S2), providing insight into the correlation directionality of each component across multiple timepoints (Fig. 3b). The top 10 metabolites in feed components (coefficient-sum ≥ 0.044) identified are presented in Fig. 3c. These metabolites had negative correlation coefficient-sum values with respect to the final titer, indicating their detrimental effect on IgG production when present in higher residual concentrations. Specifically, glutamate (Glu) showed the strongest negative correlation with the final titer, followed by leucine (Leu), valine (Val), threonine (Thr), aspartate (Asp), isoleucine (Ile), tryptophan (Trp), lysine (Lys), ornithine (Orn), and phenylalanine (Phe). Most of these are amino acids, included in feed, except ornithine, which is only present in the basal medium.

### Model-guided flux analysis to explore metabolic differences between low and high GY concentrations versus the optimum GY concentration in CHO cell cultures

In order to investigate the effects of different GY concentrations in feed media on cellular responses during the cultures, we conducted ecFBA of *i*CHO2291 on phases 6–7 which shows the most distinct SGR and Qp under various conditions. Note that calculated specific rates of metabolites and IgG as well as SGRs were used as inputs for ecFBA (“Materials and methods” section); the flux of each reaction was assigned with an averaged flux of replicates to represent a condition-specific flux state. Since the best culture performance was observed in the feed medium containing 0.5 × GY (Fig. 1b), we wondered whether the 0.5 × GY condition would have unique metabolic characteristics compared to other GY conditions. To elaborate on this, we compared the 0.5 × GY (mid-GY concentration) with 0.125 × GY (lower GY concentration) or 1.0 × GY (higher GY concentration), respectively.

**Fig. 4:**
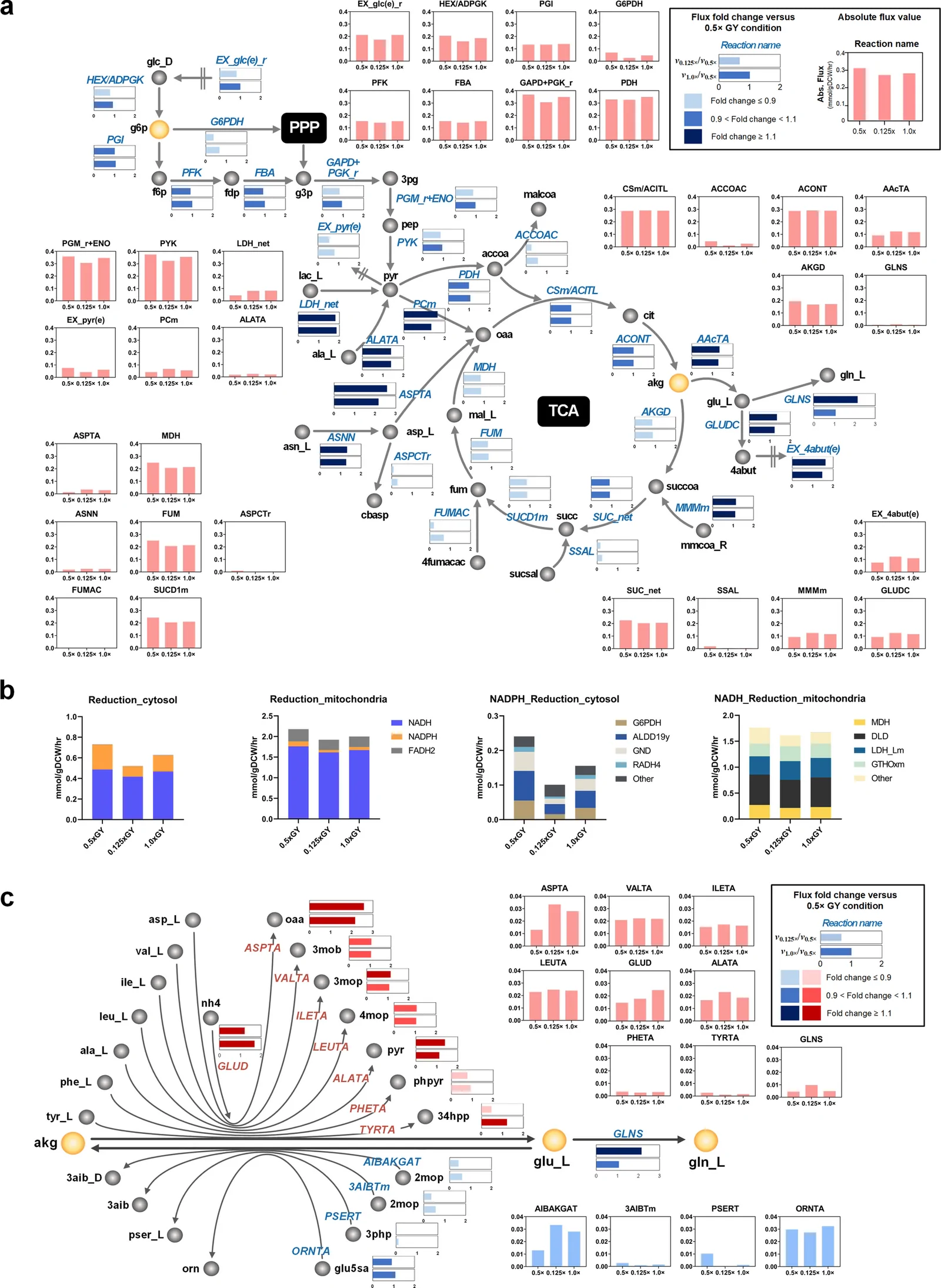
Metabolic flux comparison of 0.5 × GY control condition with lower (0.125 ×) and higher (1.0 ×) GY concentration conditions in noticeable pathways between days 6 and 7: (**a**) central carbon metabolism, (**b**) cytosolic and mitochondrial reduction of redox cofactors including NADH, NADPH, and reduced flavin adenine dinucleotide (FADH2), and **c** alpha-ketoglutarate (akg) consumption by amino acid transferases. Gray sphere indicates metabolites with their name adjacent to them. Meanwhile, noteworthy metabolites that lead to a change in flux trend are yellow spheres. Reactions are described as arrows, and their name abbreviations are written in blue italics, except the glutamate synthesis reactions which are in red. Flux fold change (*FC*) values compared to 0.5 × GY condition from each comparison set are represented as double horizontal bars, indicating a comparison of flux between 0.125 × GY and 1.0 × GY conditions. The absolute flux values of each reaction are shown in a vertical bar chart. Cytosolic NADPH reduction and mitochondrial NADH reduction, each comprise of multiple reactions are segmented according to the values of the top 4 large flux reactions and the others. Full name of metabolites and reactions are documented in the supplementary information

From the ecFBA results, among 6190 reactions counting forward and backward separately, we only selected the reactions with more than 20% higher flux values in the 0.5 × condition compared to 0.125 × or 1.0 × GY, resulting in 271 and 238 reactions sets for the subsequent comparative analysis of 0.5 × GY *vs*. 0.125 × GY and 0.5 × GY *vs*. 1.0 × GY, respectively. We identified the repeatedly existing reactions in two comparison sets: fatty acid synthesis, cholesterol metabolism, nucleotide biosynthesis, tRNA charging, valine/leucine/isoleucine metabolism, pentose phosphate pathway (PPP), glycine/serine/threonine metabolism, glycerophospholipid metabolism, glycolysis/glucogenesis, and tyrosine metabolism. Most of these reactions are associated with central carbon metabolism, tRNA charging, and cholesterol metabolism, taking part in the production of energy cofactors necessary for cell proliferation and biosynthesis. It is surprising that these metabolisms were attenuated in both the 0.125 × GY condition (considered GY-depleted) and the 1.0 × GY condition (GY-excessive) (Fig. 2). This observation supports the superior titer and growth characteristics of the 0.5 × GY condition. Specifically, the 0.5 × GY condition had higher flux distributions from glucose into the PPP, which mediated by glucose-6-phosphate dehydrogenase (G6PDH) (Fig. 4a). This flux augmentation supported a higher reduction of nicotinamide adenine dinucleotide phosphate (NADP +), with flux values reaching 165% and 50% higher levels in the 0.5 × GY condition compared to the 0.125 × and 1.0 × GY conditions, respectively, while maintaining a comparable glucose uptake rate across all conditions (Fig. 4b). Similarly, the 0.5 × GY had relatively intensified flux of 2-oxoglutarate dehydrogenase (AKGD) in the tricarboxylic acid (TCA) cycle, while the TCA cycle was supplied with a similar amount of carbon sources in all three conditions. This was due to the lower consumption of alpha-ketoglutarate (akg) through amino acid transaminases (AAcTAs) in the 0.5 × GY, which was 35% and 29% lower flux values than the 0.125 × GY and 1.0 × GY conditions, respectively. Notably, the AKGD in the 0.5 × GY condition was shown to have 16% and 13% higher flux compared to the 0.125 × GY and 1.0 × GY conditions, respectively. Higher flux trends of 0.5 × GY were maintained throughout TCA cycle until malate dehydrogenase (MDH), generating 26% and 17% more of mitochondrial NADH (Fig. 4b, c). The higher flux in both PPP and TCA cycle enabled a sufficient reduction of redox cofactors, consequently, cells in the 0.5 × GY condition produced 139% and 55% more reduced NADP + (i.e., NADPH) in total compared to the 0.125 × GY and 1.0 × GY conditions, respectively. Additionally, there was a 9% and 5% increase in total fluxes generating reduced mitochondrial nicotinamide adenine dinucleotide (i.e., NADH) in 0.5 × GY. These findings reflect an increased ATP generation of cells in 0.5 × GY condition in accord with elevated cell growth and protein production in the 0.5 × GY condition.

Since MVDA highlighted the most statistically significant impact of amino acids such as Glu, Leu, Val, Thr, Asp, Ile, Trp, Lys, Orn, and Phe on the final titer, we conducted an in-depth analysis of their metabolism to mechanistically understand how and why these particular amino acids contribute to enhancing the IgG production. Most of all, Glu exhibited the most significant negative correlation with the final titer, consequently, we scrutinized the flux of reactions involved in Glu metabolism. Their reactions can be grouped into three major categories: those that convert Glu to produce glutamine (Gln), 4-Aminobutanoate (4abut), and cellular protein, respectively. Biosynthesis of 4abut takes up 77 ~ 81% of total available Glu, but the entire synthesized 4abut was secreted extracellularly (Fig. 4a). On the other hand, protein synthesis consumed only 1 ~ 3% of Glu. Approximately 4 ~ 6% of Glu was converted into Gln through glutamine synthetase (GLNS), demonstrating the significant role of GLNS as a major source of Gln (Fig. 4a, b). However, the synthesis of Gln from Glu seemed to overrun the demand, since the oversupply of Gln in the 1.0 × GY condition, led to 50% of the disposal of total available Gln. This phenomenon was more pronounced in the 0.125 × GY condition, where 87% of the remaining Gln was secreted out. Only 5% of the supplied Gln was excreted in the 0.5 × GY condition, as it showed a relatively lower ratio of Gln synthesis to Glu synthesis (1.3) compared to the ratios observed in the 0.125 × GY (5.0) and 1.0 × GY (1.9) conditions. This observation clearly indicated that the efficient utilization of Glu in the 0.5 × GY condition, in other words, higher levels of Glu or Gln did not improve further biomass production or protein synthesis, which is in good agreement with the previous study (Ha and Lee 2014). As Glu was also identified as negatively correlated to the final titer, reducing Glu levels was considered. However, because Glu plays a versatile role in akg production as well as protein synthesis, the precursor, Gln was targeted to be reduced in media, expecting less accumulation of Glu and its decreased availability. Furthermore, lowering Gln was expected to be beneficial for enhancing culture performance by mitigating the production of ammonia resulting from its conversion into Glu (Kim et al. 2013).

Asp was also identified as having a negative impact on the final titer and is closely interconnected with Glu/Gln metabolism and TCA cycle. Asp is converted into oxaloacetic acid (Oaa) while simultaneously consuming akg to produce Glu. Additionally, Asp can be utilized for protein synthesis as well as nucleotide production (Yao et al. 2021). Among nucleotide biosynthesis reactions, Asp is converted into carbamoyl-L-aspartate (cbasp), which is used for coenzyme q10 reduction. The conversion rate of Asp into cbasp was remarkable for the 0.5 × GY condition (28%/3%/7% of total available Asp for 0.5 × GY/0.125 × GY/1.0 × GY). This efficient utilization of Asp into biomass and energy-generating cofactors may explain the higher proliferation and production observed in the 0.5 × GY condition. On the other hand, in the 0.125 × GY and 1.0 × GY conditions, a higher conversion of Asp and akg into Oaa and Glu through aspartate transaminase (ASPTA) was shown, the ASPTA flux took up 92% (0.125 × GY) and 81% (1.0 × GY) of total Asp fluxes. Although ASPTA supplied Oaa into the TCA cycle, contribution to total Oaa supplementation was trivial (4 ~ 11%) compared to mitochondrial malate dehydrogenase (MDH, 67 ~ 82%) or phosphoenolpyruvate carboxylase (PCm, 14 ~ 22%). However, ASPTA simultaneously consumes equimolar akg as it converts Asp into Oaa, contributing as the most akg-consuming reaction among AAcTAs (22% and 18% in the 0.125 × GY and 1.0 × GY) (Fig. 4c). This highlights the significant effect of akg consumption via ASPTA, which exhibited 2- to 2.3 times higher flux in the 0.125 × GY and 1.0 × GY conditions compared to the 0.5 × GY condition.

### Improvement of IgG production by modulating synergistic amino acids with the optimum GY concentration in the media

The reduction of Gln supplementation in CHO cell culture is suggested based on insights obtained from MVDA and ecFBA, intending to diminish the cellular utilization of Glu and prevent the heightened accumulation of toxic metabolites and excessive transmitter Gln at high Glu levels. The hypothesis behind this approach is that limiting the supply of Gln can restrict the availability of Glu, considering Gln as a known precursor for Glu in mammalian cells (Fomina-Yadlin et al. 2014). To do so, we carefully estimated the Gln concentration to ensure it does not adversely affect cell growth and productivity, since Gln is an essential amino acid for CHO cells and a substantial reduction in Gln levels could potentially harm cell health and protein production. Consequently, we achieved the targeted reduction by providing 2 mM of Gln instead of the originally used 6 mM.

Based on the results of the Asp/Asn analyses, we hypothesized that an excessive amount of Asp may drive the conversion of akg into Glu and subsequently reduce the reaction fluxes within the TCA cycle. To avoid this, it needs to consider reducing the Asp concentration to a certain level. As previously mentioned, Asp is a versatile component that could be utilized for the synthesis of nucleotide and coenzyme q10 (q10h2), depletion of which may result in attenuated cellular growth. Thus, the reduction of Asn in feed media was prompted by the fact that 87 ~ 96% of Asn was converted into Asp, releasing an equimolar amount of ammonia (Fig. 4a). This observation aligns with a prior study where decreasing Asn led to less ammonia accumulation without significant impact on the central carbon metabolism (McAtee Pereira et al. 2018). In addition, we further explored other amino acids associated with the AAcTA reaction and identified ornithine transaminase (ORNTA) as a key driver of increased akg supply in the 0.5 × GY condition (Fig. 4c). ORNTA catalyzes the conversion of Glu to akg and transforms Orn concurrently (Chong et al. 2010) so that reducing the Orn concentration was considered to induce more Orn and akg synthesis. Orn can be converted into two different metabolites (Figure S3): citrulline (citr_L), which eventually converts into fumarate, one of the key intermediate metabolites in the TCA cycle, and putrescine (ptrc), whose higher secretion level signifies cell apoptosis (Takao et al. 2006). This further supports the rationale for decreasing Orn levels to sustain lower AAcTA flux within the TCA cycle. Note that Orn is not included in the control feed medium. Instead, we targeted the reduction of arginine (Arg), the precursor of Orn, in the feed for validation experiments.

**Fig. 5:**
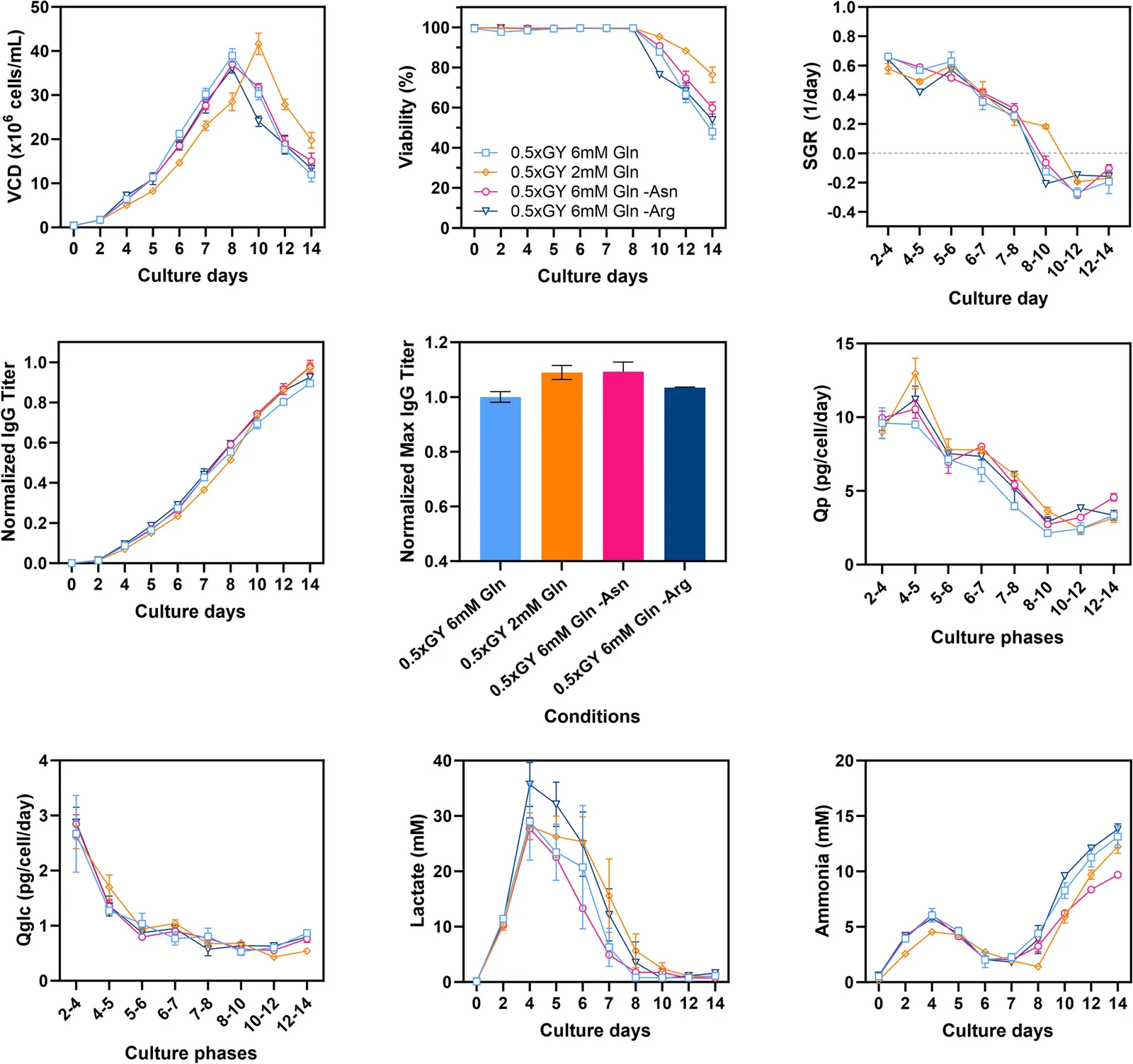
Profiles of confirmation runs for the reduction of glutamine (Gln) supplementation and for the reduction of asparagine (Asn) or arginine (Arg) in the 0.5 × GY-containing feed media. The profiles include VCD, viability, SGR, titer, maximum titer, Qp, Q_GLC_, and concentration of lactate and ammonia. Error bars represent the standard deviations of biological duplicates or triplicates. In instances where the error bars are shorter than the size of the symbol, they may not be visibly distinguishable

The concentration of each specific amino acid (Gln, Asn, and Arg) in the new feed formulation was carefully determined, ensuring that it would not induce deprivation of the target amino acids. This was assumed based on the consumption rate remaining similar to that observed in the 0.5 × GY condition with 6 mM of Gln (as a control condition). The confirmation runs were carried out, and their corresponding culture profiles are presented in Fig. 5. The reduction in Gln supplementation to 2 mM led to significantly different culture profiles during cultivation. The cells exhibited prolonged growth compared to the control condition with higher (6 mM) Gln supplementation and reached their peak VCD on day 10, achieving the highest value of 41.61 × 10^6^ cells/mL. This represented a 6.32% increase compared to the pVCD observed in the control condition, with the highest SGR particularly during culture phases 8–10. Furthermore, the cells maintained a viability of over 80% throughout the culture period, extending until day 14. As expected, this enhanced cell growth positively impacted the IgG production. The feed containing 0.5 × GY supplemented with 2 mM Gln resulted in an 8.96% increase in the final titer and a higher Qp overtime compared to the control condition. Notably, the glucose uptake rate was also efficient from the initial cell growth and remained stable throughout the culture period. Additionally, ammonia formation exhibited lower levels than in the control condition, even lower than in other conditions, while lactate accumulation was comparatively higher, although it is worth mentioning that lactate measurements had some large error bars. As we hypothesized, Gln is metabolized by cells through various pathways, including the conversion of Gln to Glu and then to αkg, which serves as a key intermediate in the TCA cycle. During this metabolic process, ammonia is produced as a byproduct. Detoxification of ammonia requires energy, diverting resources away from other vital cellular processes. Therefore, by reducing ammonia production through lower Gln supplementation, cells can allocate more energy and resources to growth and protein production. This reduction in cellular stress contributes to improved cell growth, viability, and protein production.

Next, the reduction of Asn in the feed resulted in a VCD profile that was comparable with that of the control with slightly improved viability over the course of cultivation. Interestingly, what is particularly noteworthy is that the Asn subtracted condition led to the most significant performance enhancement with a 9.22% increase in the final titer. This enhancement can be attributed to comparative cell growth after reaching pVCD on day 8, as compared to the control condition. The Qp increased consistently from culture phases 6–7 until the end of the culture. Notably, this condition exhibited lower accumulation of toxic metabolites such as lactate and ammonia in comparison to the control condition. In fact, the ammonia level was the lowest among all the conditions tested. On the other hand, the condition with Arg subtraction also showed promise, achieving a 3.4% increase in the final titer with slightly higher Qp than the control condition. However, it is worth mentioning that the cell culture experienced a significant decline after reaching pVCD, and viability dropped accordingly. Lactate production was higher in this condition compared to the others. Overall, the reduction of Arg seemed to induce an imbalanced response in the cells. Thus, further study is needed by carefully adjusting targeted amino acid levels, we can achieve a better balance of nutrients in the 0.5 × GY contained feed medium. Overall, the synergistic nutrient component in the feed media can promote healthier cell growth and expedite protein production.

## Discussion

L-Tyr is an important essential amino acid for cellular metabolism and protein production, as well as product quality in CHO cell culture. With the growing interest in process intensification within the biopharmaceutical industry (Chen et al. 2018), providing sufficient and optimal Tyr-equivalents becomes crucial for achieving the best process performance during cultivating cells in enriched or highly concentrated media conditions. In fed-batch, the pH of the enriched or concentrated feed media is often altered to increase the solubility of free Tyr, but this creates the need for complex control strategies to minimize pH spikes or gradients during feed additions and mitigate the risk of precipitation. This approach also leads to an increase in osmolality through multiple pH shifts and is not suitable for enhancing solubility in perfusion media or intensifying/simplifying the culture process. To overcome the core challenges associated with free Tyr, the biopharmaceutical industry has turned to the adoption of dipeptides in cell culture media, a well-established practice known to improve CHO culture performance. Several studies reported about the Tyr-containing dipeptide: different types/forms and their boosting properties, and cellular uptakes. To the best of our knowledge, our study is the first report to present relatively detailed investigations on the effects of different concentrations of Tyr-containing dipeptide, GY, in feed media on CHO cell culture performance, especially on the final titer, and their metabolic differences by the cells. In this study, we identified the best-performed GY concentration for IgG1 production in CHO-K1 cells under the given culture conditions and demonstrated the role of adjustable media components like Gln and Asn as synergistic additives, confirming our hypothesis through confirmation experiments. Notably, Asn and Asp are well-known amino acids that can convert into each other, thus complementing each other in metabolic pathways. Typically, Asn is converted into Asp via ASNN, releasing an equivalent amount of ammonia (Duarte et al. 2014). However, our validation results from Asp supplementation in combination with Asn subtraction did not show the reducing effect of Asn. This suggests that the addition of Asp may have contributed to increased Asn levels in cellular metabolism (Kirsch et al. 2022). Hence, for further analysis, the combined effect of subtracting both Asn and Asp could be considered. This approach may lead to a more pronounced reduction effect on Asn/Asp metabolism for further enhancing the titer.

In addition, we investigated the metabolic pathways of other MVDA-derived candidates (Leu, Val, Thr, Ile, Trp, Lys, Orn, and Phe, in Fig. 3b, c), following a similar analysis conducted for Glu and Asp. However, these metabolites did not play a significant role in other parts of metabolism, nor did they exhibit any distinct trends in condition-specific uptake and utilization flux, except for biomass synthesis. Therefore, we decided to exclude them from the list of targeted amino acids for additional validation. We classified them into two groups based on their utilization characteristics for further elucidation (Park et al. 2018; Pereira et al. 2018). Group 1 included amino acids (Leu, Val, Ile Thr, and Phe) with certain utilization pathways that were followed by the significant secretion of subsequent metabolites, and group 2 comprised amino acids (Lys, Trp) without explicit routes, except for biomass and protein synthesis. Leu was found to be related to cholesterol synthesis as it was turned into  the cholesterol precursor, 4-methyl-2-oxopentanoate (4mop), via leucine transaminase (LEUTA), but this synthesis was contingent on the elevated biomass demand, thereby the remaining 4mop were secreted out unproductively. Val and Ile exhibited a similar utilization state in condition-specific biomass synthesis, with 30% of the remaining residuals being secreted out. The rest were converted into either 3-methyl-2-oxobutanoate (3mob) or 3-methyl-2-oxopentanoate (3mop), eventually metabolizing into succinyl-CoA within the TCA cycle. Their fluxes did not show condition-specific differences, varying by less than 5%. The consumption of Thr remained similar across all conditions, fluctuating within a 7% range. However, the demand for biomass was higher ranging from 1.8- to 3.9-fold in the 0.5 × GY condition, resulting in a slight 20% reduction in the conversion path into glycine, which in turn held less significance. Phe was converted into Tyr (42 ~ 67%) or discarded after turning into phenylpyruvate (25 ~ 32%), the variance in flux primarily arose from increased Tyr consumption in the 0.5 × GY, and neither conversion pathway exhibited a clear connection to key metabolism.

The limitations in simulating the burdening effect of up-taking amino acids and conducting cleavage processes to utilize them as monomers are present with fluxes via FBA. The present analysis relies on the acquired metabolic profile alterations resulting from changes in amino acid concentrations. While changes in GY concentration have demonstrated substantial impacts on culture performance, the calculated fluxes related to GY or Tyr did not show significant changes that could be elucidated as the key events leading to performance improvements. Highlighting these changes in terms of fold-change proves challenging, as it narrowing down the process, requiring a specific search for affected fluxes. It is possible that higher concentrations of GY or increased uptake rates and release of leftover Tyr into the media could be causing some stress, but further investigation is needed to elucidate the exact underlying mechanisms. In this study, we observed that in conditions with GY concentrations exceeding that of the 0.5 × GY condition, substantial amounts of residual GY and Tyr remained in the media, implying that higher GY concentrations (1.0 × , 2.0 × GY) were metabolized and released outside the cells. However, previous studies have presented conflicting findings. For example, in murine hybridoma cells, a study indicated a significant impact of extracellular hydrolysis when investigating the utilization of L-alanyl-L-glutamine and glycyl-L-glutamine (Christie and Butler 1994). In contrast, other studies have suggested that the observed rapid clearance may result from their swift transport into the cell, rather than hydrolysis (Kang et al. 2012). Specifically, Tyr-containing dipeptides such as alanyl-L-Tyr, GY, and prolyl-L-Tyr have been examined and reported to be taken up by CHO cells and undergo intracellular cleavage before entering anabolic and catabolic pathways (Sánchez-Kopper et al. 2016). Evidently, a comprehensive understanding of how dipeptides are utilized, their mechanisms of action, and their fate within cells remains elusive.

The current CHO-GEM with ecFBA has some limitations in revealing how dipeptides are efficiently taken up and metabolized by the cell due to incomplete model coverage, with missing *k*_cat_ values for some enzymes, and limited mechanistic understanding of their intracellular metabolism and utilization. Note that the missing or unknown *k*_cat_ values in the model imply a lack of specific kinetic information about enzyme activity, making it challenging to accurately predict how dipeptides are metabolized because enzyme kinetics play a significant role in these processes. Thus, integrating amino acid transporter genes and their associated reactions into a CHO-GEM could greatly enhance its specificity and comprehensiveness. Additionally, this task can be complex as some metabolic processes linked to dipeptide metabolism, potentially resulting in their exclusion from the model. Moreover, amino acids whose concentration should be precisely controlled are the key components in feed media, but their flux analysis only relies on the catabolism into the TCA cycle or anabolism into proteins. Even if the amino acid concentration is supplied more in new formulations, some of them are pumped out by transporters, which makes it very complicated to estimate the uptake rate of amino acids. Therefore, with the conventional approach of current GEMs, we rely on experimentally derived uptake or secretion rates of amino acids to analyze subsequent effects on several pathways. Thus this analysis may not extend beyond figuring out intracellular pathway bottleneck. Owing to these limitations, choosing the right concentration of amino acids in feed media requires more experimental and analytical endeavors. In this regard, introducing a model capable of simulating changes in component uptake through transporters would allow us to accurately calculate expected metabolic states under varying amino acid conditions.

In conclusion, this study provides an in-depth investigation into the challenging amino acid supply in the feed media during CHO cell culture, highlights the need for a deeper understanding of cellular dipeptide metabolism, and demonstrates the potential of optimizing media formulations using specific concentrations of dipeptides, i.e., glycyl-L-tyrosine (GY), to enhance IgG1 production. Furthermore, the identification of amino acids (Gln and Asn) that alleviate metabolic bottlenecks emphasizes the significance of considering synergistic effects to enhance culture performance.

## Supplementary Information

Below is the link to the electronic supplementary material.

Supplementary file1 (PDF 907 KB)

## Data Availability

Most data generated or analyzed during this study has been incorporated into this manuscript and its accompanying supplementary information file.
