# Supplementary material for: Exploring metabolic effects of dipeptide feed media on CHO cell cultures by in silico model-guided flux analysis
Source: Appl Microbiol Biotechnol. 2024 Jan 16;108:123. doi: 10.1007/s00253-023-12997-0 (PMC10791731; doi:10.1007/s00253-023-12997-0)
Supplement: Supplementary file 1 [file 253_2023_12997_MOESM1_ESM.pdf]

Applied Microbiology and Biotechnology

Supplementary information

Exploring metabolic effects of dipeptide feed media on CHO cell cultures by *in silico* model-guided flux analysis

Seo-Young Park[1], Jinsung Song[1], Dong-Hyuk Choi[1], Uiseon Park[2], Hyeran Cho[2], Bee Hak Hong[2], Yaron R. Silberberg[2], Dong-Yup Lee[1,*]

[1]School of Chemical Engineering, Sungkyunkwan University, 2066 Seobu-ro, Jangan-gu, Suwon-si, Gyeonggi-do 16419, South Korea

[2]Ajinomoto Genexine Co., Ltd., CELLiST Solution Center, 70 Songdogwahak-ro, Yeonsu-gu, Incheon, South Korea

---

*Correspondence to:

Dong-Yup Lee

Tel: +82-31-290-7253; fax: +82-31-290-7272; e-mail: dongyuplee@skku.edu

Table S1 Coefficients and coefficients-sum during the post-feeding phase (days 4-14) from BLM-PLS analysis.

Coefficient	Day 4	Day 5	Day 6	Day 7	Day 8	Day 10	Day 12	Day 14	Coef. sum
**Glu**	0.009354	0.010637	0.009046	-0.011671	-0.009962	-0.010962	-0.011601	-0.012961	-0.0571
**Leu**	0.002228	-0.006680	0.004521	-0.011397	-0.013037	-0.008309	-0.009883	-0.013420	-0.0560
**Val**	0.003094	-0.007485	0.000744	-0.011577	-0.007720	-0.008549	-0.009359	-0.013157	-0.0503
**Thr**	0.003276	-0.006324	0.001176	-0.012714	-0.006737	-0.007501	-0.008352	-0.012431	-0.0477
**Asp**	0.012441	0.001422	0.00790	-0.012541	-0.006546	-0.010657	-0.013175	-0.016756	-0.0472
**Ile**	0.002040	-0.007119	0.003908	-0.009385	-0.006702	-0.008277	-0.009330	-0.012931	-0.0466
**Trp**	-0.007596	-0.006408	0.001433	-0.011955	-0.011038	-0.006738	-0.006627	-0.009567	-0.0459
**Lys**	0.006545	-0.007107	0.002341	-0.012738	-0.006151	-0.006797	-0.007841	-0.012379	-0.0459
**Orn**	0.005827	-0.007293	0.003758	-0.010768	-0.017803	-0.014411	-0.009511	-0.013375	-0.0455
**Phe**	-0.00606	-0.008396	-0.004011	-0.011329	-0.005672	-0.007838	-0.007815	-0.011579	-0.0442
**Ser**	0.005437	-0.005430	-0.000733	-0.011886	-0.008511	-0.010789	-0.007999	-0.012785	-0.0439
**Met**	0.000152	-0.008198	-0.005655	-0.010384	-0.006008	-0.007635	-0.007603	-0.012241	-0.0438
**His**	-0.001931	-0.006983	-0.005486	-0.009993	-0.005176	-0.007648	-0.008367	-0.012364	-0.0435
**Ala**	-0.001537	-0.010792	-0.007174	-0.009946	-0.011502	-0.010382	-0.013891	-0.012619	-0.0368
**Arg**	-0.001330	-0.008577	-0.005695	-0.006205	-0.004988	-0.007355	-0.005897	-0.011215	-0.0356
**NH4**	0.009966	0.011917	0.012620	-0.008455	-0.005247	-0.005994	-0.009323	-0.006553	-0.0355
**Tau**	-0.002099	-0.002128	0.006143	-0.000594	-0.006557	-0.001327	-0.010686	-0.016453	-0.0342
**Pro**	0.000251	-0.009223	-0.003321	-0.007633	-0.005685	-0.006942	-0.005956	-0.011729	-0.0319
**Cys**	0.014034	0.006436	0.009781	0.009111	0.005964	-0.013371	-0.011315	-0.013460	-0.0241
**Gln**	0.003793	0.003733	0.015242	-0.009210	-0.011267	-0.005982	-0.004774	-0.006475	-0.0224
**Asn**	0.008428	-0.001657	0.010767	0.002095	0.005683	0.000166	-0.004767	-0.014241	-0.0190
**Lac**	-0.002584	-0.005693	-0.003846	0.002147	-0.004641	-0.007501	-0.012015	-0.018766	-0.0187

Table S2 Metabolites with VIP scores exceeding 1.00 during the post-feeding phase (days 4-14) based on BLM-PLS analysis.

VIP score	Day 4	Day 5	Day 6	Day 7	Day 8	Day 10	Day 12	Day 14
**Glu**	0.7161	0.7641	0.6380	1.0540	1.3001	1.3862	1.4109	1.4615
**Leu**	0.5640	0.4778	0.3755	1.3467	1.3490	1.2708	1.3476	1.4686
**Val**	0.6335	0.5423	0.3132	1.3174	1.2484	1.2853	1.3067	1.4416
**Thr**	0.7120	0.4844	0.4956	1.4140	1.2080	1.2408	1.2734	1.4202
**Asp**	1.0009	0.1022	0.5848	1.1445	1.1888	1.3739	1.4134	1.5271
**Ile**	0.5649	0.5073	0.3221	1.2511	1.2069	1.2733	1.3222	1.4512
**Trp**	0.6589	0.4754	0.3982	1.3883	1.3052	1.1898	1.2220	1.3022
**Lys**	0.9030	0.5461	0.6279	1.3909	1.1761	1.2222	1.2398	1.4158
**Orn**	0.9007	0.5565	0.6232	0.8060	1.4448	1.2685	0.7663	1.1655
**Phe**	0.4420	0.5972	0.4260	1.3314	1.1662	1.2346	1.2622	1.3693
**Ser**	0.6369	0.3856	0.2035	1.2428	1.1895	1.2852	0.9002	1.2568
**Met**	0.4095	0.5821	0.4000	1.2800	1.1743	1.2314	1.1985	1.3448
**His**	0.3731	0.4991	0.4077	1.2203	1.1471	1.2248	1.2524	1.3733
**Ala**	0.6409	0.7917	0.7105	0.7062	0.8324	1.1591	1.380	1.3422
**Arg**	0.5020	0.6081	0.4082	1.0587	1.1229	1.1968	1.0555	1.2685
**NH4**	0.7028	0.8565	0.8979	1.1672	1.1376	1.1752	1.3104	1.1003
**Tau**	0.2683	0.2738	0.5363	1.0279	1.1831	0.9496	1.1615	1.4508
**Pro**	0.4914	0.6523	0.2638	0.9966	1.1369	1.1824	0.9847	1.2548
**Cys**	1.0868	0.5302	0.9863	0.9775	0.7051	1.2706	1.4020	1.4659
**Gln**	0.8203	0.7097	1.0972	1.3004	1.3802	1.1742	1.1059	1.1861
**Asn**	0.6413	0.2391	0.9282	0.5788	0.751	0.9524	1.0263	1.5039
**Lac**	0.4912	0.5080	0.3917	0.8997	0.7097	0.7844	0.8883	1.3323

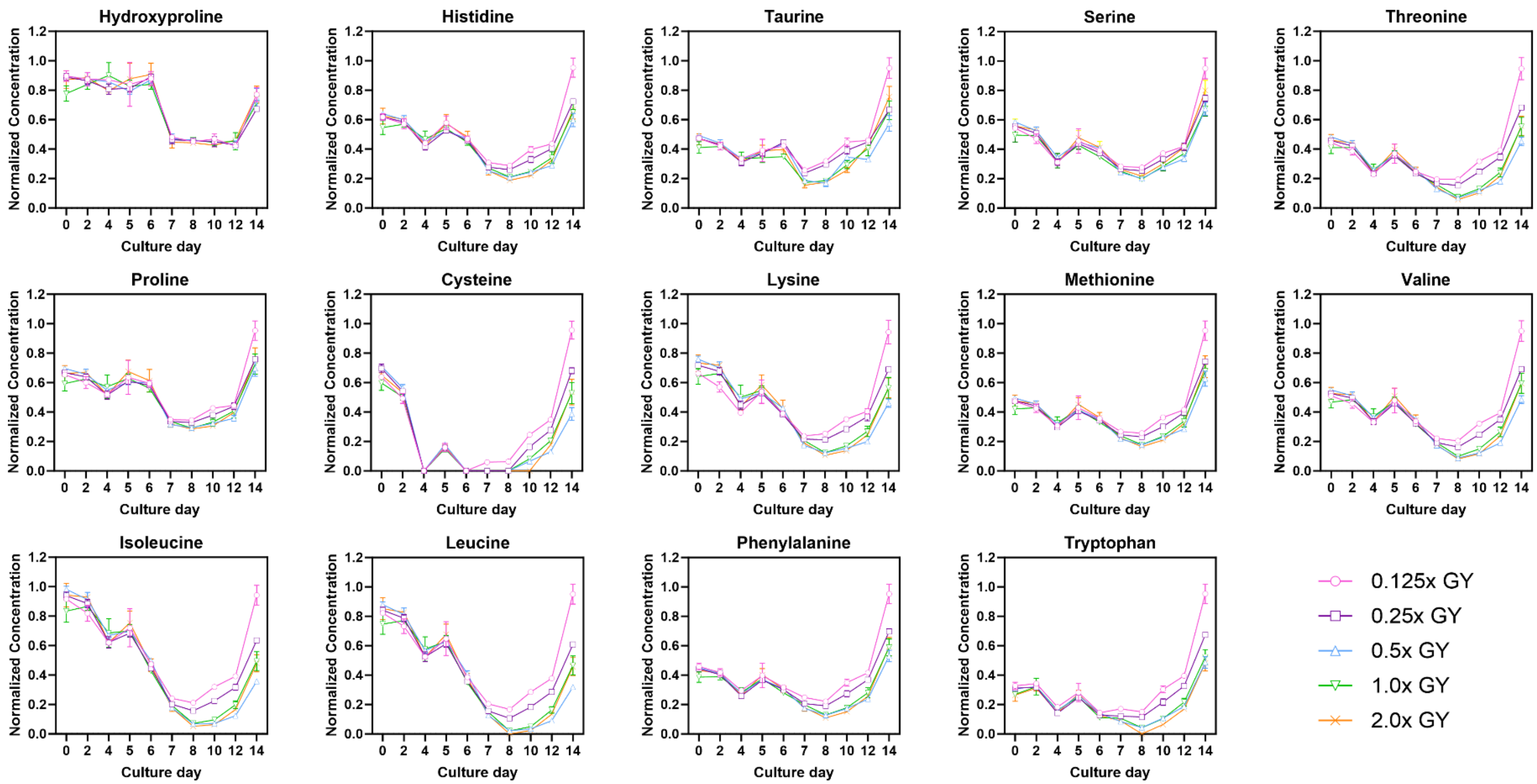

Fig. S1 Profiles of residual concentration of amino acids not shown in Fig. 2.

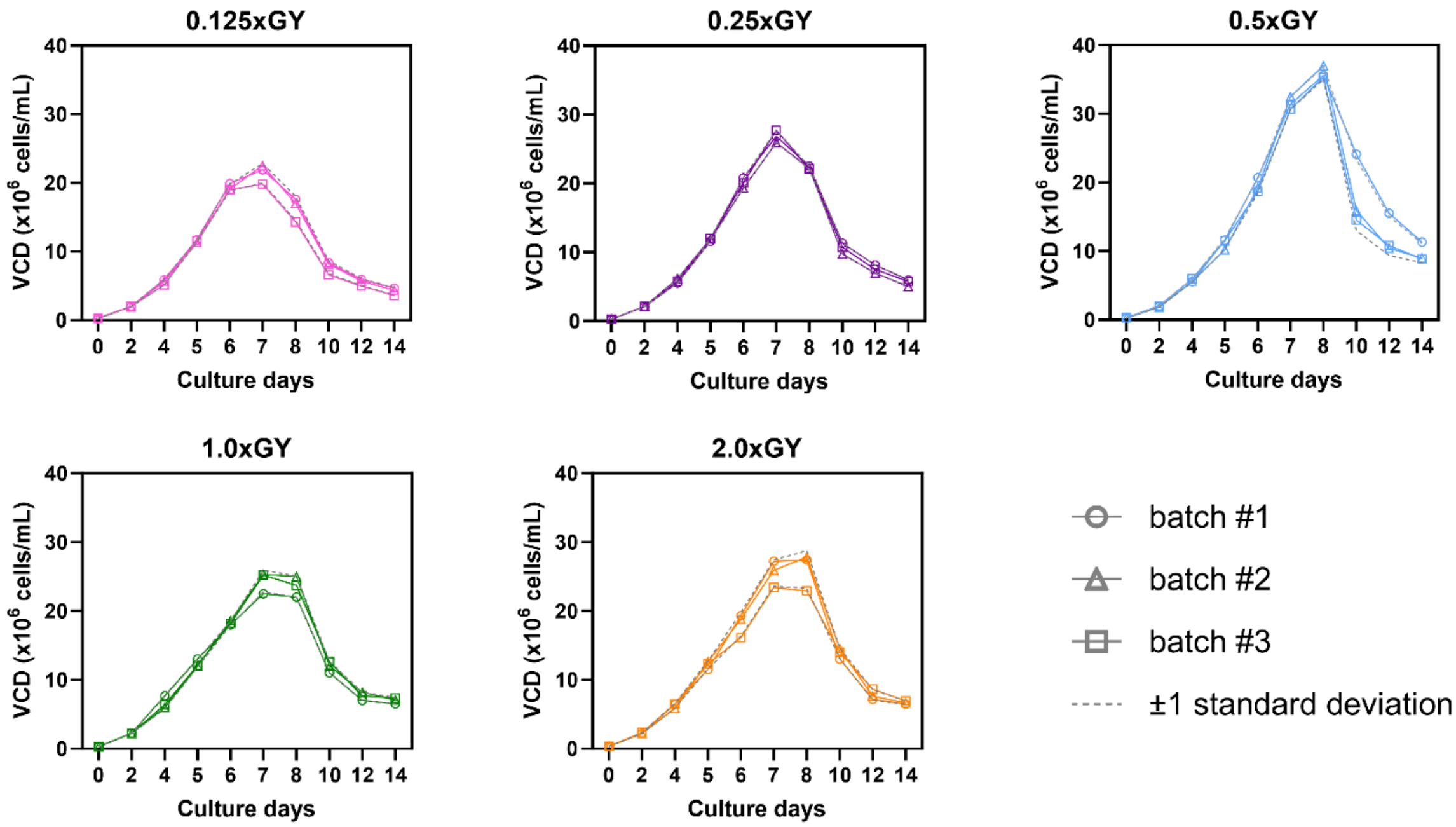

Fig. S2 Identification of outlier batch based on the evaluation of VCD profiles from each GY concentration condition's batch dataset.

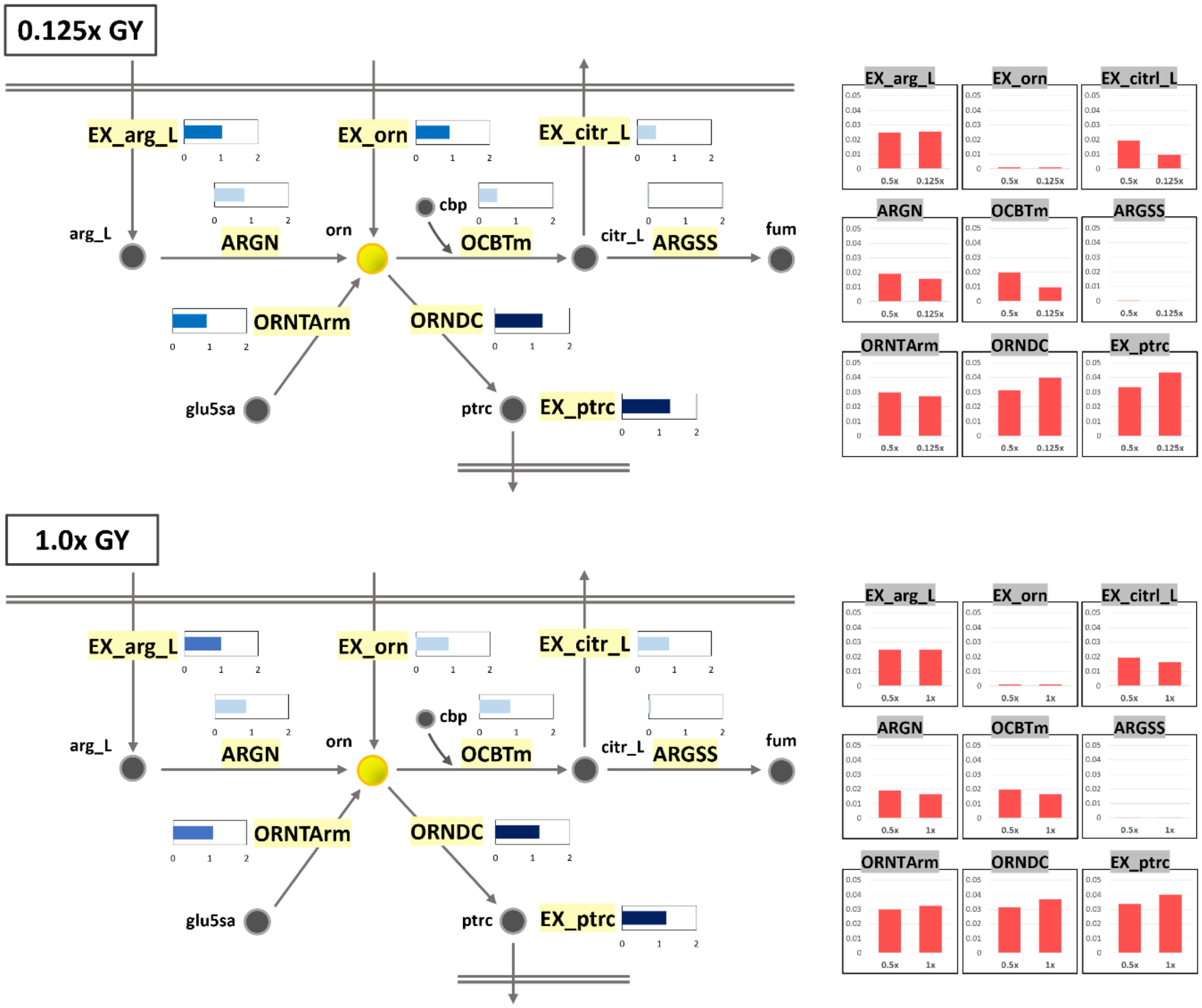

Fig. S3 Metabolic flux comparison of the optimum (0.5×) GY condition with lower (0.125×) and higher (1.0×) GY conditions in ornithine (orn) pathway.

Abbreviation Full name of metabolites and reactions for Fig. 4.

Abbreviation for metabolite listed alphabetically:

2mop, 2-Methyl-3-oxopropanoate; **34hpp**, 3-(4-Hydroxyphenyl)pyruvate; **3aib**, L-3-Amino-isobutanoate; **3aib_D**, D-3-Amino-isobutanoate; **3mob**, 3-Methyl-2-oxobutanoate; **3mop**, (S)-3-Methyl-2-oxopentanoate; **3pg**, 3-Phospho-D-glycerate; **3php**, 3-Phosphohydroxypyruvate; **4abut**, 4-Aminobutanoate; **4fumacac**, 4-Fumarylacetoacetate; **4mop**, 4-Methyl-2-oxopentanoate; **accoa**, Acetyl-CoA; **akg**, 2-Oxoglutarate; **ala_L**, L-Alanine; **asn_L**, L-Asparagine; **asp_L**, L-Aspartate; **cbasp**, N-Carbamoyl-L-aspartate; **cit**, Citrate; **f6p**, D-Fructose 6-phosphate; **fdp**, D-Fructose 1,6-bisphosphate; **fum**, Fumarate; **g3p**, Glyceraldehyde 3-phosphate; **g6p**, D-Glucose 6-phosphate; **glc_D**, D-Glucose; **gln_L**, L-Glutamine; **glu_L**, L-Glutamate; **glu5sa**, L-Glutamate 5-semialdehyde; **ile_L**, L-Isoleucine; **lac_L**, L-Lactate; **leu_L**, L-Leucine; **mal_L**, L-Malate; **malcoa**, Malonyl CoA; **mmcoa_R**, (R)-Methylmalonyl-CoA; **oaa**, Oxaloacetate; **orn**, Ornithine; **pep**, Phosphoenolpyruvate; **phe_L**, L-Phenylalanine; **phpyr**, Phenylpyruvate; **pser_L**, O-Phospho-L-serine; **pyr**, Pyruvate; **succ**, Succinate; **succoa**, Succinyl-CoA; **sucsal**, Succinic semialdehyde; **tyr_L**, L-Tyrosine; **val_L**, L-Valine;

Abbreviation for reactions listed alphabetically:

3AIBTm, L-3-aminoisobutyrate transaminase (mitochondrial); **AAcTA**, Amino acid transaminase; **ACCOAC**, Acetyl-CoA carboxylase; **ACONT**, Aconitate hydratase; **AIBAKGAT_r**, (R)-3-Amino-2-methylpropanoate; **AKGD**, 2-oxoglutarate dehydrogenase; **ALATA**, L-alanine transaminase; **ALDD19y**, Aldehyde dehydrogenase-NADP; **ASNN**, L-asparaginase; **ASPCTr**, Aspartate carbamoyl transferase; **ASPTA**, Aspartate transaminase; **CSm/ACITL**, Citrate synthase (mitochondria)/ATP-Citrate lyase; **DLD**, Dihydrolipoamide dehydrogenase; **EX_4abut(e)**, 4-Aminobutanoate exchange; **EX_glc(e)_r**, D-glucose exchange; **Ex_glu_L(e)_r**, L-glutamate exchange; **EX_pyr(e)**, Pyruvate exchange; **FBA**, Fructose-bisphosphate aldolase; **FUM**, Fumarase; **FUMAC**, Fumarylacetoacetase; **G6PDH**, Glucose 6-phosphate dehydrogenase; **GAPD+PGK_r**, Glyceraldehyde-3-phosphate dehydrogenase, Phosphoglycerate kinase (lumped); **GLNS**, Glutamine synthetase; **GLUD**, Glutamate dehydrogenase; **GLUDC**, Glutamate decarboxylase; **GND**, Phosphogluconate dehydrogenase; **GTHOxm**, Glutathione NAD+ oxidoreductase (mitochondrial); **HEX/ADPGK**, Hexokinase/ADP-dependent glucokinase; **ILETA**, Isoleucine transaminase; **LDH_Lm**, Lactate dehydrogenase (mitochondria); **LDH_net**, Lactate dehydrogenase (net reaction); **LEUTA**, Leucine transaminase; **MDH**, Malate dehydrogenase; **MMMm**, Methylmalonyl-CoA mutase (mitochondria); **ORNTArm_r**, Ornithine transaminase reversible (mitochondria); **PCm**, Pyruvate carboxylase (mitochondria); **PDH**, Pyruvate carboxylase (mitochondria); **PEPCK**, Phosphoenolpyruvate carboxykinase; **PFK**, Phosphofructokinase; **PGI**, Glucose-6-phosphate isomerase; **PGM_r+ENO**, Phosphoglycerate mutase, Enolase (lumped); **PHETA**, Phenylalanine transaminase; **PSERT**, Phosphoserine transaminase; **PYK**, Pyruvate kinase; **RADH4**, Retinal dehydrogenase; **SSAL**, Succinate-semialdehyde dehydrogenase reversible (mitochondrial); **SUC_net**, Succinate-CoA ligase (GDP-forming); **SUCD1m**, Succinate dehydrogenase (mitochondria); **TYRTA**, Tyrosine transaminase; **VALTA**, Valine transaminase;
