# Supplementary material for: Exploring metabolic effects of dipeptide feed media on CHO cell cultures by in silico model-guided flux analysis
Source: Appl Microbiol Biotechnol. 2024 Jan 16;108(1):123. doi: 10.1007/s00253-023-12997-0 (PMC10791731; doi:10.1007/s00253-023-12997-0)
Supplement: Supplementary file 1 — Supplementary file1 (PDF 907 KB) [file 253_2023_12997_MOESM1_ESM.pdf]

**Applied Microbiology and Biotechnology**

# Supplementary information

---

Exploring metabolic effects of dipeptide feed media on CHO cell cultures by *in silico* model-guided flux analysis

Seo-Young Park[1], Jinsung Song[1], Dong-Hyuk Choi[1], Uiseon Park[2], Hyeran Cho[2], Bee Hak Hong[2], Yaron R. Silberberg[2], Dong-Yup Lee[1,*]

[1]School of Chemical Engineering, Sungkyunkwan University, 2066 Seobu-ro, Jangan-gu, Suwon-si, Gyeonggi-do 16419, South Korea

[2]Ajinomoto Genexine Co., Ltd., CELLiST Solution Center, 70 Songdogwahak-ro, Yeonsu-gu, Incheon, South Korea

---

*Correspondence to:

Dong-Yup Lee

Tel: +82-31-290-7253; fax: +82-31-290-7272; e-mail: dongyuplee@skku.edu

**Table S1** Coefficients and coefficients-sum during the post-feeding phase (days 4-14) from BLM-PLS analysis.

| Coefficient | Day 4 | Day 5 | Day 6 | Day 7 | Day 8 | Day 10 | Day 12 | Day 14 | Coef. sum |
|---|---|---|---|---|---|---|---|---|---|
| Glu | 0.009354 | 0.010637 | 0.009046 | -0.011671 | -0.009962 | -0.010962 | -0.011601 | -0.012961 | -0.0571 |
| Leu | 0.002228 | -0.006680 | 0.004521 | -0.011397 | -0.013037 | -0.008309 | -0.009883 | -0.013420 | -0.0560 |
| Val | 0.003094 | -0.007485 | 0.000744 | -0.011577 | -0.007720 | -0.008549 | -0.009359 | -0.013157 | -0.0503 |
| Thr | 0.003276 | -0.006324 | 0.001176 | -0.012714 | -0.006737 | -0.007501 | -0.008352 | -0.012431 | -0.0477 |
| Asp | 0.012441 | 0.001422 | 0.00790 | -0.012541 | -0.006546 | -0.010657 | -0.013175 | -0.016756 | -0.0472 |
| Ile | 0.002040 | -0.007119 | 0.003908 | -0.009385 | -0.006702 | -0.008277 | -0.009330 | -0.012931 | -0.0466 |
| Trp | -0.007596 | -0.006408 | 0.001433 | -0.011955 | -0.011038 | -0.006738 | -0.006627 | -0.009567 | -0.0459 |
| Lys | 0.006545 | -0.007107 | 0.002341 | -0.012738 | -0.006151 | -0.006797 | -0.007841 | -0.012379 | -0.0459 |
| Orn | 0.005827 | -0.007293 | 0.003758 | -0.010768 | -0.017803 | -0.014411 | -0.009511 | -0.013375 | -0.0455 |
| Phe | -0.00606 | -0.008396 | -0.004011 | -0.011329 | -0.005672 | -0.007838 | -0.007815 | -0.011579 | -0.0442 |
| Ser | 0.005437 | -0.005430 | -0.000733 | -0.011886 | -0.008511 | -0.010789 | -0.007999 | -0.012785 | -0.0439 |
| Met | 0.000152 | -0.008198 | -0.005655 | -0.010384 | -0.006008 | -0.007635 | -0.007603 | -0.012241 | -0.0438 |
| His | -0.001931 | -0.006983 | -0.005486 | -0.009993 | -0.005176 | -0.007648 | -0.008367 | -0.012364 | -0.0435 |
| Ala | -0.001537 | -0.010792 | -0.007174 | -0.009946 | -0.011502 | -0.010382 | -0.013891 | -0.012619 | -0.0368 |
| Arg | -0.001330 | -0.008577 | -0.005695 | -0.006205 | -0.004988 | -0.007355 | -0.005897 | -0.011215 | -0.0356 |
| NH4 | 0.009966 | 0.011917 | 0.012620 | -0.008455 | -0.005247 | -0.005994 | -0.009323 | -0.006553 | -0.0355 |
| Tau | -0.002099 | -0.002128 | 0.006143 | -0.000594 | -0.006557 | -0.001327 | -0.010686 | -0.016453 | -0.0342 |
| Pro | 0.000251 | -0.009223 | -0.003321 | -0.007633 | -0.005685 | -0.006942 | -0.005956 | -0.011729 | -0.0319 |
| Cys | 0.014034 | 0.006436 | 0.009781 | 0.009111 | 0.005964 | -0.013371 | -0.011315 | -0.013460 | -0.0241 |
| Gln | 0.003793 | 0.003733 | 0.015242 | -0.009210 | -0.011267 | -0.005982 | -0.004774 | -0.006475 | -0.0224 |
| Asn | 0.008428 | -0.001657 | 0.010767 | 0.002095 | 0.005683 | 0.000166 | -0.004767 | -0.014241 | -0.0190 |
| Lac | -0.002584 | -0.005693 | -0.003846 | 0.002147 | -0.004641 | -0.007501 | -0.012015 | -0.018766 | -0.0187 |

**Table S2** Metabolites with VIP scores exceeding 1.00 during the post-feeding phase (days 4-14) based on BLM-PLS analysis.

| VIP score | Day 4 | Day 5 | Day 6 | Day 7 | Day 8 | Day 10 | Day 12 | Day 14 |
|---|---|---|---|---|---|---|---|---|
| **Glu** | 0.7161 | 0.7641 | 0.6380 | 1.0540 | 1.3001 | 1.3862 | 1.4109 | 1.4615 |
| **Leu** | 0.5640 | 0.4778 | 0.3755 | 1.3467 | 1.3490 | 1.2708 | 1.3476 | 1.4686 |
| **Val** | 0.6335 | 0.5423 | 0.3132 | 1.3174 | 1.2484 | 1.2853 | 1.3067 | 1.4416 |
| **Thr** | 0.7120 | 0.4844 | 0.4956 | 1.4140 | 1.2080 | 1.2408 | 1.2734 | 1.4202 |
| **Asp** | 1.0009 | 0.1022 | 0.5848 | 1.1445 | 1.1888 | 1.3739 | 1.4134 | 1.5271 |
| **Ile** | 0.5649 | 0.5073 | 0.3221 | 1.2511 | 1.2069 | 1.2733 | 1.3222 | 1.4512 |
| **Trp** | 0.6589 | 0.4754 | 0.3982 | 1.3883 | 1.3052 | 1.1898 | 1.2220 | 1.3022 |
| **Lys** | 0.9030 | 0.5461 | 0.6279 | 1.3909 | 1.1761 | 1.2222 | 1.2398 | 1.4158 |
| **Orn** | 0.9007 | 0.5565 | 0.6232 | 0.8060 | 1.4448 | 1.2685 | 0.7663 | 1.1655 |
| **Phe** | 0.4420 | 0.5972 | 0.4260 | 1.3314 | 1.1662 | 1.2346 | 1.2622 | 1.3693 |
| **Ser** | 0.6369 | 0.3856 | 0.2035 | 1.2428 | 1.1895 | 1.2852 | 0.9002 | 1.2568 |
| **Met** | 0.4095 | 0.5821 | 0.4000 | 1.2800 | 1.1743 | 1.2314 | 1.1985 | 1.3448 |
| **His** | 0.3731 | 0.4991 | 0.4077 | 1.2203 | 1.1471 | 1.2248 | 1.2524 | 1.3733 |
| **Ala** | 0.6409 | 0.7917 | 0.7105 | 0.7062 | 0.8324 | 1.1591 | 1.380 | 1.3422 |
| **Arg** | 0.5020 | 0.6081 | 0.4082 | 1.0587 | 1.1229 | 1.1968 | 1.0555 | 1.2685 |
| **NH4** | 0.7028 | 0.8565 | 0.8979 | 1.1672 | 1.1376 | 1.1752 | 1.3104 | 1.1003 |
| **Tau** | 0.2683 | 0.2738 | 0.5363 | 1.0279 | 1.1831 | 0.9496 | 1.1615 | 1.4508 |
| **Pro** | 0.4914 | 0.6523 | 0.2638 | 0.9966 | 1.1369 | 1.1824 | 0.9847 | 1.2548 |
| **Cys** | 1.0868 | 0.5302 | 0.9863 | 0.9775 | 0.7051 | 1.2706 | 1.4020 | 1.4659 |
| **Gln** | 0.8203 | 0.7097 | 1.0972 | 1.3004 | 1.3802 | 1.1742 | 1.1059 | 1.1861 |
| **Asn** | 0.6413 | 0.2391 | 0.9282 | 0.5788 | 0.751 | 0.9524 | 1.0263 | 1.5039 |
| **Lac** | 0.4912 | 0.5080 | 0.3917 | 0.8997 | 0.7097 | 0.7844 | 0.8883 | 1.3323 |

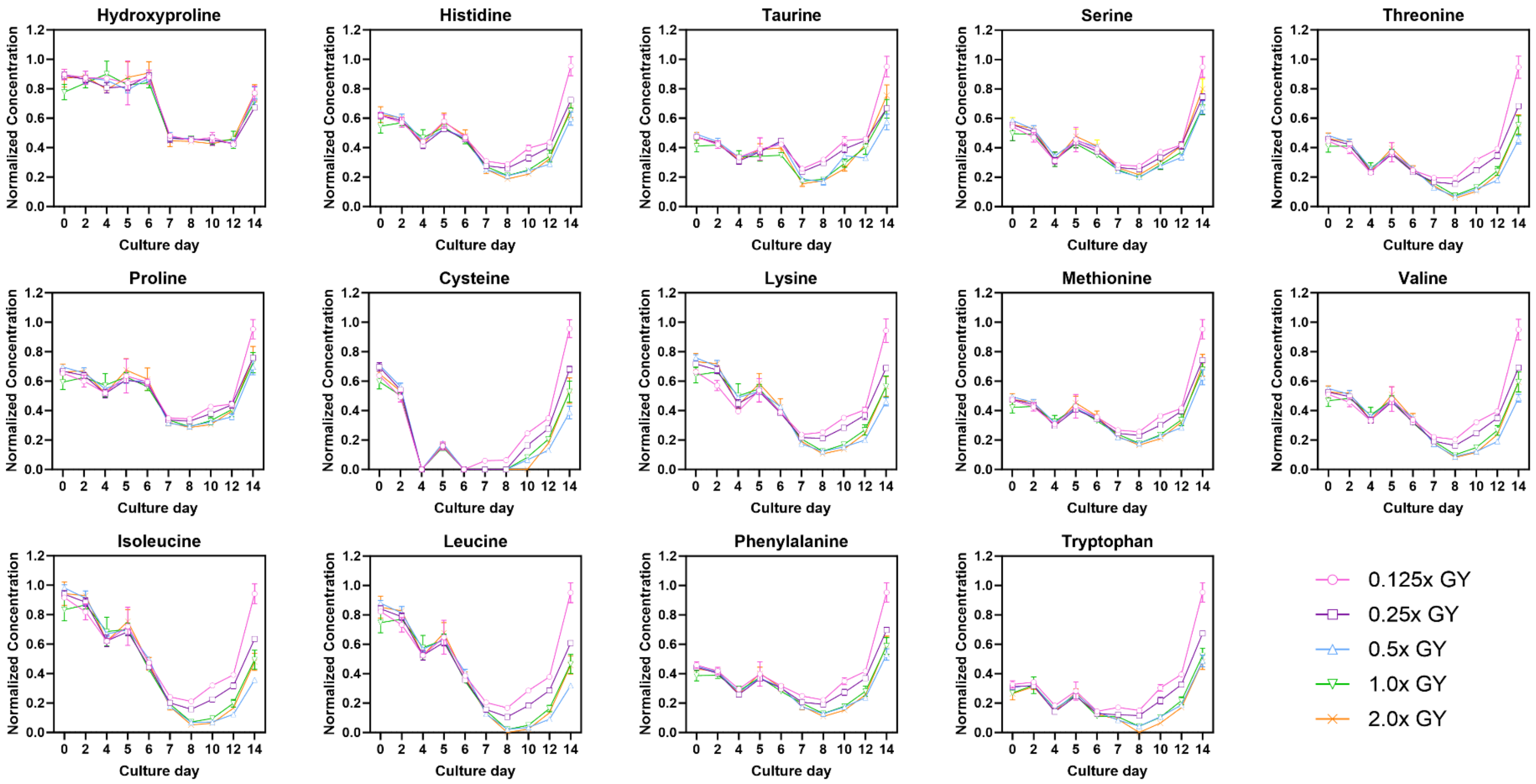


**Fig. S1** Profiles of residual concentration of amino acids not shown in Fig. 2.

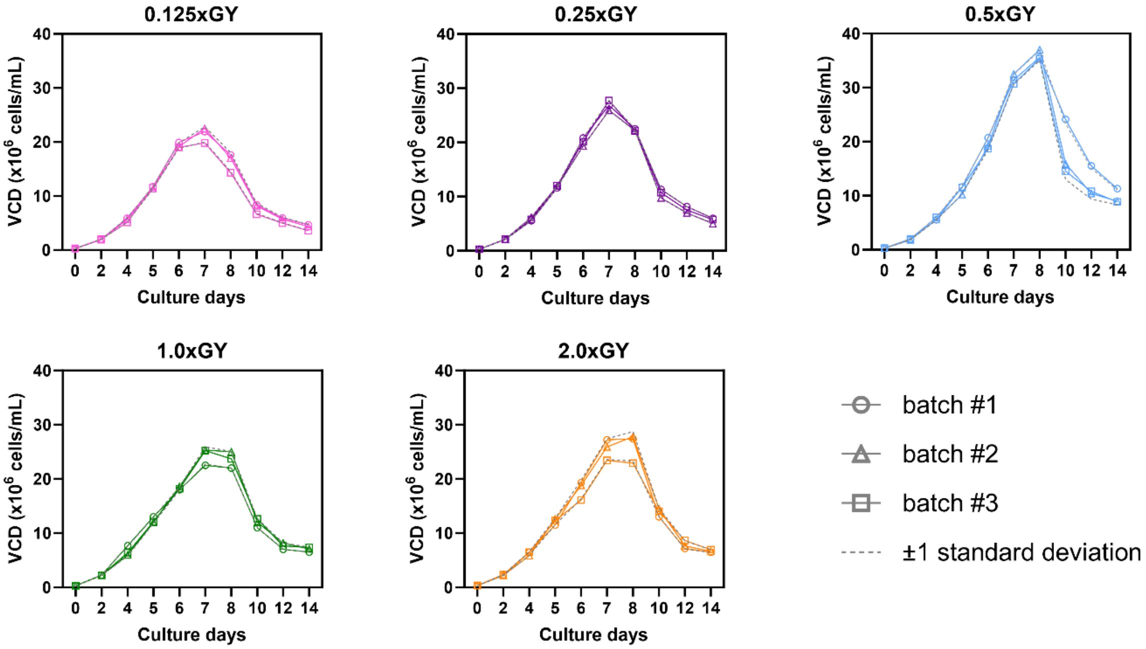


**Fig. S2** Identification of outlier batch based on the evaluation of VCD profiles from each GY concentration condition's batch dataset.

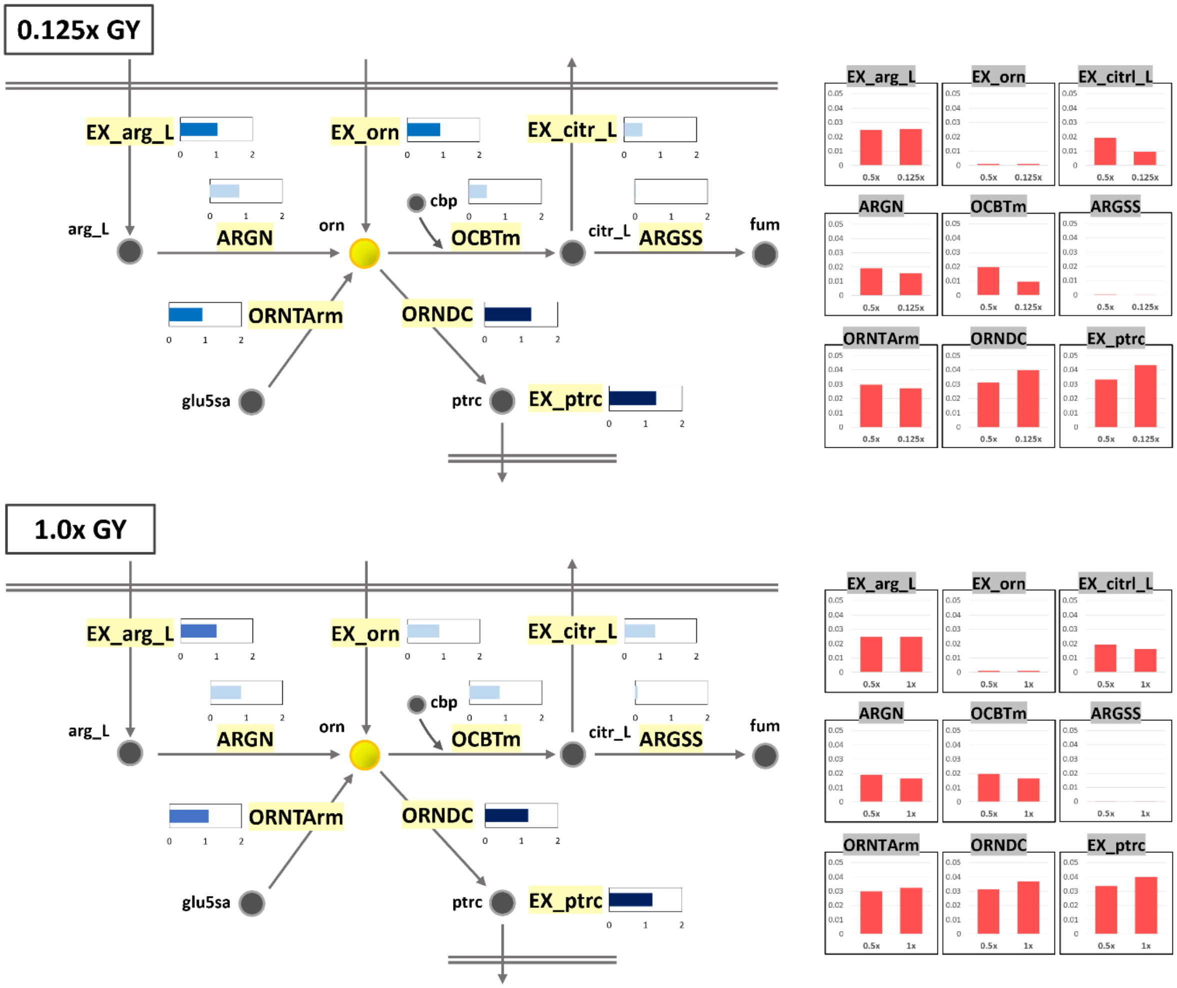


**Fig. S3** Metabolic flux comparison of the optimum (0.5×) GY condition with lower (0.125×) and higher (1.0×) GY conditions in ornithine (orn) pathway.

**Abbreviation** Full name of metabolites and reactions for Fig. 4.

**Abbreviation for metabolite listed alphabetically:**

**2mop**, 2-Methyl-3-oxopropanoate; **34hpp**, 3-(4-Hydroxyphenyl)pyruvate; **3aib**, L-3-Amino-isobutanoate; **3aib_D**, D-3-Amino-isobutanoate; **3mob**, 3-Methyl-2-oxobutanoate; **3mop**, (S)-3-Methyl-2-oxopentanoate; **3pg**, 3-Phospho-D-glycerate; **3php**, 3-Phosphohydroxypyruvate; **4abut**, 4-Aminobutanoate; **4fumacac**, 4-Fumarylacetoacetate; **4mop**, 4-Methyl-2-oxopentanoate; **accoa**, Acetyl-CoA; **akg**, 2-Oxoglutarate; **ala_L**, L-Alanine; **asn_L**, L-Asparagine; **asp_L**, L-Aspartate; **cbasp**, N-Carbamoyl-L-aspartate; **cit**, Citrate; **f6p**, D-Fructose 6-phosphate; **fdp**, D-Fructose 1,6-bisphosphate; **fum**, Fumarate; **g3p**, Glyceraldehyde 3-phosphate; **g6p**, D-Glucose 6-phosphate; **glc_D**, D-Glucose; **gln_L**, L-Glutamine; **glu_L**, L-Glutamate; **glu5sa**, L-Glutamate 5-semialdehyde; **ile_L**, L-Isoleucine; **lac_L**, L-Lactate; **leu_L**, L-Leucine; **mal_L**, L-Malate; **malcoa**, Malonyl CoA; **mmcoa_R**, (R)-Methylmalonyl-CoA; **oaa**, Oxaloacetate; **orn**, Ornithine; **pep**, Phosphoenolpyruvate; **phe_L**, L-Phenylalanine; **phpyr**, Phenylpyruvate; **pser_L**, O-Phospho-L-serine; **pyr**, Pyruvate; **succ**, Succinate; **succoa**, Succinyl-CoA; **sucsal**, Succinic semialdehyde; **tyr_L**, L-Tyrosine; **val_L**, L-Valine;

**Abbreviation for reactions listed alphabetically:**

**3AIBTm**, L-3-aminoisobutyrate transaminase (mitochondrial); **AAcTA**, Amino acid transaminase; **ACCOAC**, Acetyl-CoA carboxylase; **ACONT**, Aconitate hydratase; **AIBAKGAT_r**, (R)-3-Amino-2-methylpropanoate; **AKGD**, 2-oxoglutarate dehydrogenase; **ALATA**, L-alanine transaminase; **ALDD19y**, Aldehyde dehydrogenase-NADP; **ASNN**, L-asparaginase; **ASPCTr**, Aspartate carbamoyl transferase; **ASPTA**, Aspartate transaminase; **CSm/ACITL**, Citrate synthase (mitochondria)/ATP-Citrate lyase; **DLD**, Dihydrolipoamide dehydrogenase; **EX_4abut(e)**, 4-Aminobutanoate exchange; **EX_glc(e)_r**, D-glucose exchange; **Ex_glu_L(e)_r**, L-glutamate exchange; **EX_pyr(e)**, Pyruvate exchange; **FBA**, Fructose-bisphosphate aldolase; **FUM**, Fumarase; **FUMAC**, Fumarylacetoacetase; **G6PDH**, Glucose 6-phosphate dehydrogenase; **GAPD+PGK_r**, Glyceraldehyde-3-phosphate dehydrogenase, Phosphoglycerate kinase (lumped); **GLNS**, Glutamine synthetase; **GLUD**, Glutamate dehydrogenase; **GLUDC**, Glutamate decarboxylase; **GND**, Phosphogluconate dehydrogenase; **GTHOxm**, Glutathione NAD+ oxidoreductase (mitochondrial); **HEX/ADPGK**, Hexokinase/ADP-dependent glucokinase; **ILETA**, Isoleucine transaminase; **LDH_Lm**, Lactate dehydrogenase (mitochondria); **LDH_net**, Lactate dehydrogenase (net reaction); **LEUTA**, Leucine transaminase; **MDH**, Malate dehydrogenase; **MMMm**, Methylmalonyl-CoA mutase (mitochondria); **ORNTArm_r**, Ornithine transaminase reversible (mitochondria); **PCm**, Pyruvate carboxylase (mitochondria); **PDH**, Pyruvate carboxylase (mitochondria); **PEPCK**, Phosphoenolpyruvate carboxykinase; **PFK**, Phosphofructokinase; **PGI**, Glucose-6-phosphate isomerase; **PGM_r+ENO**, Phosphoglycerate mutase, Enolase (lumped); **PHETA**, Phenylalanine transaminase; **PSERT**, Phosphoserine transaminase; **PYK**, Pyruvate kinase; **RADH4**, Retinal dehydrogenase; **SSAL**, Succinate-semialdehyde dehydrogenase reversible (mitochondrial); **SUC_net**, Succinate-CoA ligase (GDP-forming); **SUCD1m**, Succinate dehydrogenase (mitochondria); **TYRTA**, Tyrosine transaminase; **VALTA**, Valine transaminase;
